# Deformity, erosion, lesion, tumor, and parasite (DELT) anomalies in fish communities of the Chesapeake Bay watershed, USA: a regional assessment and potential landscape drivers

**DOI:** 10.1007/s10661-025-14412-9

**Published:** 2025-08-08

**Authors:** Sara E. Breitmeyer, Paul McLaughlin, Vicki S. Blazer, Gregory B. Noe, Kelly L. Smalling, Timothy Wertz, Tyler Wagner

**Affiliations:** 1 Pennsylvania Water Science Center, U.S. Geological Survey, Downingtown, PA 19335 USA; 2 Pennsylvania Cooperative Fish and Wildlife Research Unit, The Pennsylvania State University, University Park, PA USA; 3 Eastern Ecological Science Center Leetown Research Laboratory, U.S. Geological Survey, Kearneysville, WV 25430 USA; 4 Florence Bascom Geoscience Center, U.S. Geological Survey, Dover, DE 19901 USA; 5 New Jersey Water Science Center, U.S. Geological Survey, Lawrenceville, NJ 08648 USA; 6 Bureau of Clean Water, Pennsylvania Department of Environmental Protection, Harrisburg, PA 17101 USA; 7 U.S. Geological Survey, Pennsylvania Cooperative Fish and Wildlife Research Unit, University Park, PA USA

**Keywords:** Biological assessment, Fish health, Freshwater ecosystems, Watershed-scale drivers

## Abstract

**Abstract:**

Fish diseases in freshwater ecosystems pose significant ecological and socioeconomic challenges, yet monitoring them in wild populations is complex due to interactions between pathogens, hosts, and environmental conditions. We examine the prevalence and watershed-scale landscape drivers of external deformity, erosion, lesion, tumor, and parasite (DELT) anomalies in 57 riverine fish species using a large dataset (577,266 individuals collected 2008–2019) from the Chesapeake Bay watershed that originated from state and federal agencies. Overall, DELT prevalence was low (1.4%), but was higher in larger, longer-lived species, including Channel Catfish (*Ictalurus punctatus*) (18.9%), Rock Bass (*Ambloplites rupestris*) (7.6%), Smallmouth Bass (*Micropterus dolomieu*) (7.3%), Brown Bullhead (*Ameiurus nebulosus*) (5.6%), and Yellow Bullhead (*Ameiurus natalis*) (5.1%), signifying their potential as regional environmental health indicators. Spatial analysis indicated warmer temperatures increased the estimated probability of DELT occurrence, whereas higher precipitation often mitigated the probability of DELT occurrence. Conservation strategies (e.g., best management practices) had mixed effectiveness in reducing DELT occurrence probability across agricultural and urban landscapes. Across the landscape, various drivers, including harvested forest, impervious land, and pesticide use, influenced DELT occurrence probability differently across species. However, uncertainty remains partly due to low prevalence and variability in sampling methods across agencies. Despite low overall prevalence, DELT occurrence is a rapid fish health indicator. Future research could emphasize species-specific responses and longitudinal studies that incorporate life stages and health indicators. Understanding these intricate, multi-scale interactions is vital for effective monitoring, conservation, and adaptive management of freshwater ecosystems.

**Graphical abstract:**

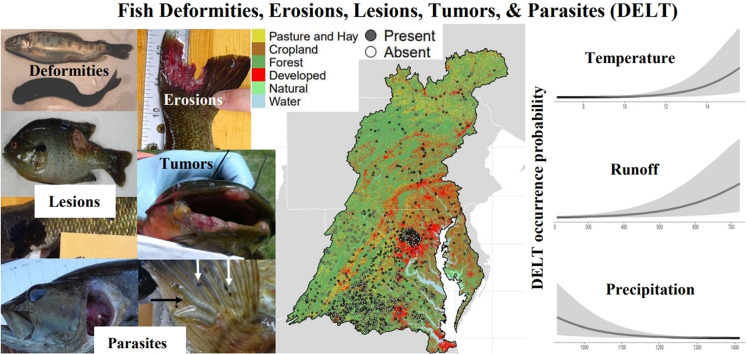

**Supplementary Information:**

The online version contains supplementary material available at 10.1007/s10661-025-14412-9.

## Introduction

Fish have long served as sentinels for aquatic ecosystem health, contributing to our understanding of the long-term impacts of anthropogenic stressors and land management practices on aquatic environments (Harris, 1995; Adams, 2002; Pinna et al., 2023; USEPA, 2016). Numerous multi-metric indices, such as fish indices of biotic integrity (IBIs; Karr, 1981), have been developed to assess ecosystem health. The IBI fish community assessment metric commonly includes the proportion of individuals with disease, tumors, fin damage, or other abnormalities but also considers other factors, such as species richness and composition. The health of fish communities or specific fish species has also been recognized as an important indicator of ecosystem health and bioindicators of fish health range from the organism to the molecular level (Adams, 2002; Blazer et al., 2014; Kroon et al., 2017).

One fish health indicator similar to the metric used in the IBI is the DELT (deformity, erosion, lesion, tumor, parasite) index, which documents and compares the occurrence of visible external abnormalities (Sanders et al., 1999; Vidal, 2008; Simon & Burskey, 2016). Fish DELT serve as valuable indicators in ecological health assessments, such as disease monitoring and tracking water quality. For instance, a study of Bluntnose Minnow (*Pimephales notatus*) in a Michigan Area of Concern stream revealed a high prevalence of DELT (occurring in 70% of individuals) alongside internal abnormalities, including gonads and livers that were black, ruptured, and decreased in thickness (Simon & Burskey, 2016). Furthermore, research in Illinois rivers has linked increased observations of DELT in upstream fish populations to urban runoff (DeBoer et al., 2024).

However, there are challenges in evaluating DELT as an indicator of environmental quality. The deficiency of historical data and restricted spatial extent complicate efforts to assess temporal and spatial prevalence and identify potential relationships to environmental conditions. Additionally, there is a lack of uniformity in DELT methodology across state and federal agencies and researchers. Observations of DELT cannot identify causes (e.g., infectious or noninfectious) or the risk factors associated with those causes (e.g., contaminants, water quality, or environmental stressors). Nevertheless, DELT observations may identify watersheds, portions of watersheds, or specific species that require more in-depth health analyses and can be combined with anthropogenic factor and land management practice data to investigate potential local or regional drivers of aquatic ecosystem health.

Efforts to restore water quality and improve fisheries in the Chesapeake Bay has resulted in the continued implementation of best management practices (BMPs) on agricultural and urban lands (Chesapeake Bay Program, 2025b, c). These BMPs consist of a variety of structural implementations (e.g., waste containment, streambank fencing, riparian buffers, wet ponds, stormwater basins) and management actions (e.g., crop rotation, conservation tillage) aimed at reducing nitrogen, phosphorus, and sediment loads to rivers and streams within the Chesapeake Bay watershed. The potential of BMPs to provide co-benefits in the form of reducing anthropogenic contaminants, disease, and hormones has been observed (Richkus et al., 2016; Guardian et al., 2021; Gordon et al., 2023; Smalling et al., 2021); however, the overall effects of BMPs on fish health are still largely unknown.

In the broader field of landscape ecology, relating spatial patterns to ecosystem processes and understanding relevant scales for measurement has been identified as a critical area of research (Turner, 2005; Soranno et al., 2010). In aquatic systems, for example, a landscape limnology perspective has been proposed that places well-studied ecosystems into a fully connected landscape mosaic, which allows managers to extrapolate results to poorly studied systems within a similar mosaic. By effectively quantifying and managing uncertainty, Bayesian inference is identified as a robust approach to integrate different data sources and models and has been used successfully in previous efforts that provide insight on water resource management within the Chesapeake Bay watershed and the broader United States (US) (Soranno et al., 2010; Biggs et al., 2009; Wagner et al., 2022; Smalling et al., 2023).

Leveraging a meticulously combined and robust dataset, we adopt a similar landscape perspective and Bayesian inference in this study. We present what is, to the best of our knowledge, the most comprehensive evaluation of DELT anomaly prevalence (in terms of number of species and observations) over a large spatial extent. Our overall aim is to help inform current and future land and aquatic management strategies by identifying the most at-risk species and the potential landscape-scale drivers of DELT in the Chesapeake Bay watershed. The specific objectives of this study are to (1) estimate species-specific and overall (i.e., population-average, across all species) probabilities of DELT occurrence; (2) identify potential ecological drivers of both overall and species-specific fish DELT by examining associations with a range of landscape predictors, while accounting for potential sources of variation introduced by different sampling agencies and ecological regions; and (3) identify potential limitations and knowledge gained to inform future data collection and monitoring strategies.

## Methods

### Study area

The Chesapeake Bay watershed partially encompasses six states (Delaware, Maryland, New York, Pennsylvania, Virginia, and West Virginia) and the District of Columbia in the northeastern US and has a total area of approximately 165,700 km^2^ (Chesapeake Bay Program, 2025a). It is the first US estuary targeted for restoration as an adaptively managed, integrated watershed and ecosystem. Aggregated ecoregions include the Coastal Plains, Northern Appalachians, Southern Appalachians Northwest, and Southern Appalachians Piedmont (U.S. Environmental Protection Agency, 2010).

**Fig. 1:**
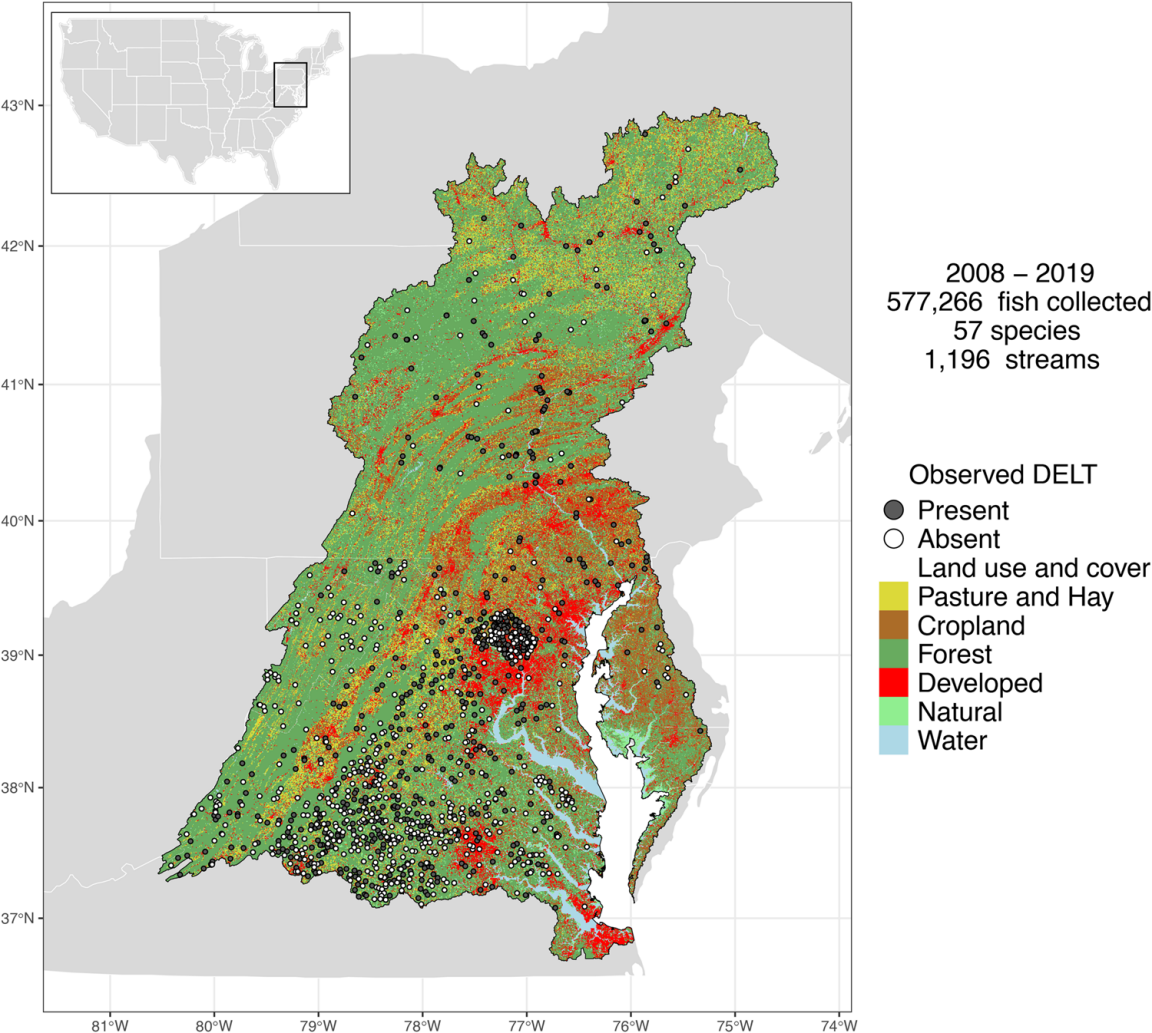
Fish collection streams (*n*, 1196) for 57 species within the Chesapeake Bay watershed, USA (black outline). Grey circles indicate the presence of observed DELT and white circles indicate its absence, in at least one individual fish at each stream. Major land use and land cover was obtained from Chesapeake Bay Program (2023). State outlines and US map were obtained from the R system R maps package (R Core Team, 2024; Becker et al., 2023). The Chesapeake Bay watershed shape outline was obtained from the Chesapeake Bay Program’s website (private member 2023)

National Hydrography Dataset Plus (NHDPlus) v2.1 flowline COMIDs (a code that uniquely identifies individual stream reaches) (McKay et al., 2012) were acquired and visually verified utilizing geographic coordinate points of stream sites where fish were collected. The COMID was used as the base spatial unit for analyses. Collection sites with available DELT data included 1196 unique non-tidally influenced streams (i.e., COMIDs), located within nine level-3 ecoregions in the Chesapeake Bay watershed (Fig. A1). The median total upstream watershed drainage area of streams was 14.6 km^2^ (range, 0.02−67,101 km^2^), and the median stream Strahler order was 2 (range, 1−7). The upstream watershed land use of sample streams had a median of approximately 16% agricultural (range, 0−77%), 7% developed (range, 0−96%), and 60% forested land (range, 1−100%)—similar proportions compared to all streams within the Chesapeake Bay watershed (Figs. 1 and A2).

### Fish collection and DELT assessment

Fishes were collected by national, state, and local agencies that utilize standardized, community-based fish collection protocols within their respective area of responsibility (Fig. A1 and Table A1). Although agencies had collected fish between 1989 to 2019, the DELT data in this study were restricted between 2008 to 2019, to more closely represent the temporal record of the majority of landscape predictors. The common length and maximum year lifespan of the top species exhibiting DELT were retrieved from https://www.fishbase.se. The percentage of the 577,266 individual fish observed for DELT, by season, slightly varied for each collection agency (Table A2). Overall, roughly 84% of individuals observed for DELT were collected in the summer (June through September) and 15% were collected in the fall (October through January). Most collection agencies selected primary sites randomly to achieve a broad-scale assessment. However, some sites were targeted to answer specific management questions (e.g., cause and effect, change in status, or protected use assessment).

**Fig. 2:**
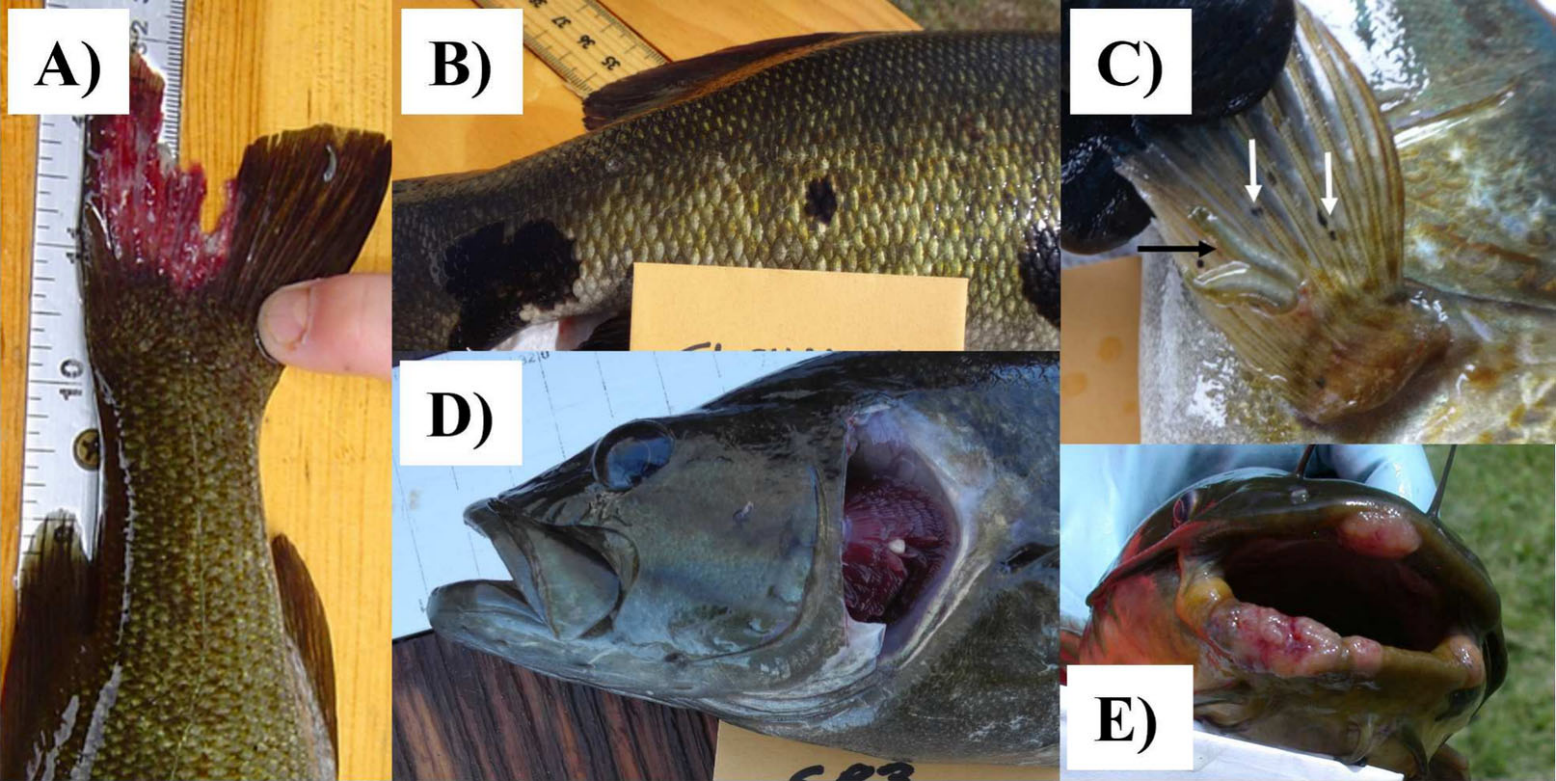
Various species of field-collected freshwater fish displaying examples of DELT including **A** an eroded fin, **B** melanistic areas, **C** parasitic leeches (black arrow) and black spots (trematode, white arrows), **D** a cyst (on gill), and **E** tumors

Fish sampling for each agency was designed to collect a representative sample of the overall fish community and characterize most species present (Table A1). Most fish species collected were representative of resident warmwater communities, with only a few species representative of cool-water communities (e.g., Creek Chub (*Semotilus atromaculatus*), Brown Trout (*Salmo trutta*)). Young-of-year individuals were excluded from analysis. After collection, individual fish were observed for specific anomalies (e.g., deformities, abrasions and erosions, fungal infections, tumors) that are typically grouped into one of the DELT anomaly categories for comparison (Fig. 2A–E and Table A3). Categories of DELT were not defined for each of the collection agencies; therefore, Pennsylvania Department of Environmental Protection (PADEP) descriptions (Table A3) were utilized to establish an alignment with (i.e., crosswalk) the data for all collection agencies (Table A4).

Differences existed across the collection agency protocols for recording DELT (Tables A1 and A4). For instance, while most agencies included external parasites (such as leeches or anchor worms), the US Environmental Protection Agency (EPA) did not. Conversely, most agencies did not evaluate gill condition; however, PADEP did. To facilitate a regional analysis while acknowledging the inherent differences in DELT recording protocols among collection agencies, specific anomalies were aggregated to presence/absence of DELT for each individual fish (i.e., if an individual fish had any anomaly it was categorized as having a DELT). This simplification allowed for meaningful spatial comparisons at a broad scale, effectively addressing the challenges posed by differing DELT recording protocols across collection agencies.

### Landscape data

The landscape predictors were gathered in Windows 10 Enterprise, version 21H2 with 64-bit ISO and the R system utilizing the nhdplusTools and sbtools packages (R Core Team, 2024; Blodgett & Johnson, 2023; Winslow et al., 2016). Landscape predictors were chosen based on stressor attributes relating to DELT in the literature and fetched from comprehensive datasets of NHDPlus v2.1 stream attributes in the Chesapeake Bay watershed and the continental US (Gordon et al., 2017; Chesapeake Bay Program, 2023; Wieczorek et al., 2018; Jones et al., 2021). A variety of predictors were chosen and included those representing habitat alteration, water quality (organic pollutants and heavy metals), industrial and urban point source pollution, nutrient enrichment, BMP implementation, altered flow regimes, sediment transport, temperature, and geology (Yeom et al., 2009; Jensen, 2023; Vidal, 2008; Maloney et al., 2021; Norton et al., 2002; Noe et al., 2020) (Table A5).

The predictors representing BMP implementation included no tillage (also known as zero-tillage) and conservation tillage (also known as reduced tillage), which are implemented to help reduce agricultural sediment and nutrient loads (Chesapeake Bay Program, 2022). In addition, two other predictors representing BMP implementation included United States Department of Agriculture (USDA) modeled estimations of sediment and nitrogen loss from agricultural fields due to a suite of USDA National Resource Conservation Service (NRCS) BMPs. The USDA NRCS BMPs considered for both estimated sediment and nitrogen loss included structural practices for controlling water and wind erosion (e.g., overland and concentrated flow control, edge-of-field buffering and filtering, and wind erosion control practices) and reduced tillage using either no tillage or mulch tillage (Table A5, refer to ag sed red BMP and ag TN red BMP definitions for specific structural practices; Wieczorek et al., 2018; United States Department of Agriculture, 2012). In addition to these BMPs, the estimated nitrogen loss also considered the appropriate timing, method (e.g., incorporated or banding/foliar/spot treatment), and rates of total nitrogen application (United States Department of Agriculture, 2012).

If landscape predictors were available across multiple years (e.g., air temperature and precipitation), for each COMID, a single mean value (calculated from annual averages) was paired with DELT data. Summary statistics for landscape predictors varied across DELT collection streams (Table A6). To assess the influence of the complete drainage basin on DELT, we paired each stream segment (COMID) of individual fish with a single static value for each landscape predictor that represented the total accumulated upstream watershed area.

#### Spatial framework

In studies of large spatial extent that span broad gradients in landscape features, such as this study, it is important to consider how multiple spatial scales influence ecosystem dynamics, how the hierarchical organization of freshwater ecosystems impact study results, and to use regional variables (e.g., ecoregion) to classify freshwater ecosystems for multi-ecosystem management (Soranno et al., 2010; Poiani et al., 2000; Higgins et al., 2005; Herlihy & Sifneos, 2008). Following methods in Soranno et al. (2010), a patch-mosaic landscape framework provided the foundation for the study streams as they interact with freshwater, terrestrial, and human landscapes (Table A5). The patch-mosaic model considers rivers as freshwater ecosystem patches that are each embedded in a terrestrial and human mosaic. The freshwater ecosystem patch characteristics (e.g., physical, chemical, and biological predictors) were organized into mosaic categories that represent (1) patch connectivity that allows for the movement of material and organisms (e.g., precipitation, runoff, groundwater, and drainage network) and (2) terrestrial and human patch contexts that drive water chemistry and species richness (e.g, national policy, geology, land use, and soils). The mosaic sub-categories for freshwater, terrestrial, and human patch characteristics were also each organized by spatial scale/hierarchy (from regional to local), illustrating processes managed at multiple spatial scales (e.g., national level policy versus local land management).

### Statistical modeling

Bayesian hierarchical logistic regression models were used to evaluate the effects of landscape characteristics on the occurrence of DELT because of their robust approach to data integration and successful application in water resource management (Soranno et al., 2010; Biggs et al., 2009; Wagner et al., 2022; Smalling et al., 2023). Three types of models were fitted which included ecoregion as a random effect to account for broad-scale spatial dependencies and for the sharing of information (i.e., partial pooling) to occur for ecoregions with smaller sample sizes. First, an unconditional model was fitted to data for all 57 species. The partial pooling that occurs in the hierarchical model (Gelman & Hill, 2006) allowed for all species to be included in this model, regardless of species-specific naive prevalence of DELT or sample sizes. This model did not include landscape predictors and was only used to estimate species-specific average probabilities of DELT occurrence and the overall (population-average) DELT occurrence probability. Let $y_{ijkrts}$ be the $i^{th}$ DELT observation ($y_{ijkrts}$ = 1; if DELT present; 0 otherwise) at COMID *j* by collection agency *k* in ecoregion *r* and in year *t* for species *s*. The model was as follows:

1 $$\begin{aligned} {\begin{matrix} Pr(y_{ijkrts} = 1) & = logit^{-1}(\beta _0 + \gamma _k + \phi _r + \eta _s) \\ & \gamma _k \sim N(0,\sigma ^2_{\textit{collection agency}}) \\ & \phi _r \sim N(0,\sigma ^2_{region}) \\ & \eta _s \sim N(0,\sigma ^2_{species}) \end{matrix}} \end{aligned}$$

where $\beta _0$ is the intercept and $\gamma _k$, $\phi _r$, and $\eta _s$ are random effects for the collection agency, ecoregion, and species, respectively. The random effects were assumed to be normally distributed with a mean of zero and variance $\sigma ^2_x$.

Second, a conditional model was fitted to data from all 57 species—similar to model 1—but this time including the landscape predictors, to identify which landscape predictors were correlated with DELT occurrence, in general and across all species. Let $y_{ijkrts}$ be the $i^{th}$ DELT observation ($y_{ijkrts}$ = 1; if DELT present; 0 otherwise) at COMID *j* by collection agency *k* in ecoregion *r* and in year *t* for species *s*. The model was as follows:

2 $$\begin{aligned} {\begin{matrix} Pr(y_{ijkrts} = 1) & = logit^{-1}(\beta _0 + \varvec{\beta } \varvec{X} + \gamma _k + \phi _r + \eta _s) \\ & \gamma _k \sim N(0,\sigma ^2_{\textit{collection agency}}) \\ & \phi _r \sim N(0,\sigma ^2_{region}) \\ & \eta _s \sim N(0,\sigma ^2_{species}) \end{matrix}} \end{aligned}$$

where $\beta _0$, $\gamma _k$, $\phi _r$, $\eta _s$, and $\sigma ^2_x$ are as defined above and $\varvec{\beta }$ is a vector of regression coefficients and ${\textbf {X}}$ is a matrix containing landscape predictor variables (each variable vector is standardized to a mean of 0 and standard deviation of 1 prior to analysis). A regularized horseshoe prior (Piironen & Vehtari, 2017) was used on $\varvec{\beta }$ to select among the landscape predictor variables (Tables A5 and A6) because we expected many of the estimated regression coefficients would be zero and to accommodate mulicollinearity among predictor variables (Grames & Forister, 2024). The regularized horseshoe prior has been shown to be useful for variable selection among correlated predictor variables (Weiss-Lehman et al., 2022; Şen et al., 2023; Grames & Forister, 2024). Previous studies have shown that the regularized horseshoe prior is insensitive to correlations among predictor variables < 0.90$-$0.95 (Lu and Lou, 2022; Grames & Forister, 2024). The regularized horseshoe prior was as follows:

3 $$\begin{aligned} \beta _f \sim N(0, \tau ^2 \tilde{\lambda }^2_f), \quad \tilde{\lambda }^2_f = \frac{c^2\lambda ^2_f}{c^2 + \tau ^2\lambda ^2_f} \end{aligned}$$

where $\tau$ is the global variance hyperparameter, *c* is the hyperparameter that determines the amount of shrinkage on the largest coefficients, and $\lambda _f$ is a local scale parameter that controls the amount of shrinkage applied to coefficient *f*. A diffuse normal prior is used for $\beta _0$ and half-Cauchy priors were used for the standard deviations of the normally distributed random effects.

Lastly, species-specific models were fitted to identify landscape predictors that were correlated with DELT occurrence for individual species. Species were only considered if they met the criteria of having a DELT naive prevalence $\ge$ 5% and sample size > 100. These criteria were established to ensure that there was some variation in occurrence to model (since many species had very low DELT prevalence) and that sample sizes were adequate. The species-specific model was the same as Eq. 2, except there was no species random effect included. For each landscape model fitted (overall model and species-specific model), to account for correlations among predictors, we only included those predictors with Spearman’s correlation ($\rho$) < 0.90 and estimated parameters with the regularized horseshoe prior for variable selection.

All models were fitted in a Bayesian framework using a Hamiltonian Monte Carlo sampling algorithm implemented in Stan (Carpenter et al., 2017) and using the cmdstanr package (Gabry and Češnovar, 2022) in software R (R Core Team, 2024). Convergence was assessed through visual inspection of posterior chains. All estimated parameters are reported as posterior means and corresponding 90% credible intervals (CIs)—predictor variables were considered significant if estimated 90% credible intervals did not include the value zero. We also calculated the posterior probability that the estimated effects $(\beta )$ were in the direction (positive or negative) of the posterior mean, the probability of direction (*pd*; Makowski et al., 2019). We considered predictor variables potentially biologically significant with a $pd>$ 0.90.

## Results

### Observed DELT prevalence

From 2008 through 2019, a total of 577,266 individual fish encompassing 57 species were collected at streams throughout the Chesapeake Bay watershed (Figs. 1 and A1 and Table A7). The DELT collection streams were spread throughout the Chesapeake Bay watershed, with a greater density in the southern half of the watershed (Fig. 1). At each collection stream, a median of 11 different species (range = 1−30) were collected and inspected for DELT. The total number of fish collected per species varied, with a median of 3658 individual fish per species (range = 37−163,552). Each species was collected at a median of 214 streams (range = 8−631).

In individual stream reaches (COMIDs), a median of 149 individual fish (range = 1−14,116) were collected and the naive (i.e., observed or not model-based) median DELT prevalence among all streams was 0.18%. Of the study streams, roughly half (47%) contained fish that had no observed DELT and half (53%) of the streams contained one or more fish with DELT (Fig. 1). The spatial distribution of observed DELT anomalies across the Chesapeake Bay watershed shows a correlation with land-use patterns. In the 1196 stream reaches, DELT presence was more commonly observed in the southeastern and central regions, where developed land use dominates. These areas include high concentrations of impervious surfaces, such as roads, rooftops, and parking lots, which were grouped within the broader “developed” category. In contrast, DELT anomalies were less frequent in the northern and southwestern watershed, particularly in forested and agricultural regions.

**Fig. 3:**
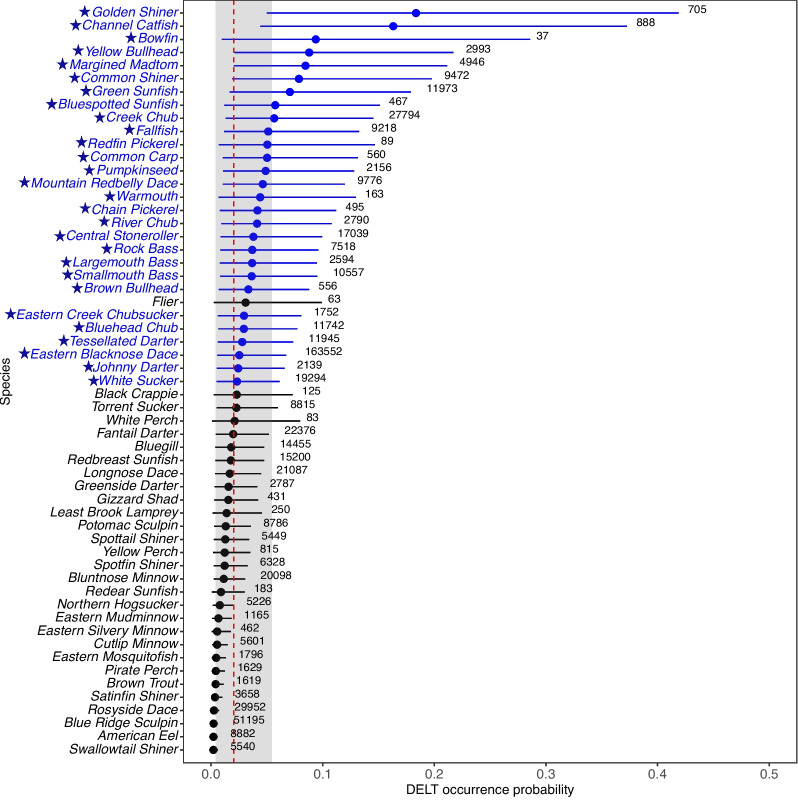
Posterior mean probability of DELT occurrence for fishes in the Chesapeake Bay watershed, USA (modeled using Eq. 1). Horizontal lines are 90% credible intervals. Species marked with a star and highlighted in blue have lower 90% credible intervals that did not include the value zero. Red dashed vertical line is the overall (across all species) average probability of DELT occurrence and shaded area is 90% credible region. Numbers are sample size (number of fish) for each species

The observed DELT prevalence across all species was 1.4% (8,121 individuals displayed DELT), and DELT occurrences for the 57 individual species ranged from 0 to 19% (Table A7). Six species exhibited a DELT prevalence greater than 5%, including the Channel Catfish (*Ictalurus punctatus*; 18.9%), Golden Shiner (*Notemigonus crysoleucas*; 8.9%), Rock Bass (*Ambloplites rupestris*; 7.6%), Smallmouth Bass (*Micropterus dolomieu*; 7.3%), Brown Bullhead (*Ameiurus nebulosus*; 5.6%), and Yellow Bullhead (*Ameiurus natalis*; 5.1%). Among the individual fish exhibiting DELT, 9.2% did not have specific DELT anomaly categories recorded to include in the overall percentages of deformities, erosions, lesions, tumors, parasites, or other due to differing collection agency protocols (e.g., sampling design based on probabilistic outcomes). For those fish that had recorded anomalies, an average of 2% had observed deformities, 13% erosions, 22% lesions, 1% tumors, 61% parasites, and 1% other (Table A4). Because the study focused on the binary presence or absence of DELT, individual fish with a DELT had at least one of these anomalies.

**Fig. 4:**
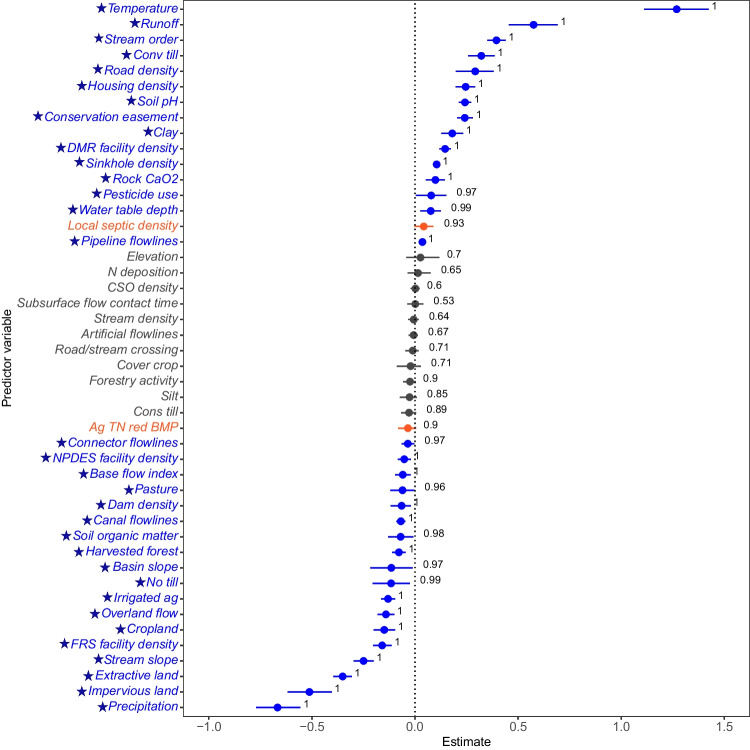
The effects of landscape predictors on the probability of DELT occurrence across all fish species sampled in the Chesapeake Bay watershed, USA (modeled using Eq. 2). Estimated effects $(\beta )$ are shown on the x-axis as posterior means (circles) and 90% credible intervals (horizontal bars). Predictors marked with a star and highlighted in blue have 90% credible intervals that do not include the value zero, predictors highlighted in orange (local septic density and Ag TN red BMP) show a high probability (*pd* > 0.90) that their estimated effects $(\beta )$ were in the direction (positive or negative) of the posterior mean, and predictors shown in grey have 90% credible intervals did not include the value zero. The posterior probability for each predictor is displayed as a numerical value

### Modeled species-specific DELT occurrence probabilities

Using the model given in Eq. 1, the population average (across all species) estimated probability of DELT occurrence was 0.02 (90% CI = 0.004, 0.054) and ranged from 0.002 (90% CI = 0.0003, 0.006) to 0.18 (90% CI = 0.05, 0.42) for the Swallowtail Shiner (*Notropis procne*) and Golden Shiner, respectively (Fig. 3). Only two species, the Golden Shiner and Channel Catfish (posterior mean occurrence probability = 0.16, 90% CI = 0.04, 0.37), had a posterior mean probability of DELT occurrence > 0.15.

**Fig. 5:**
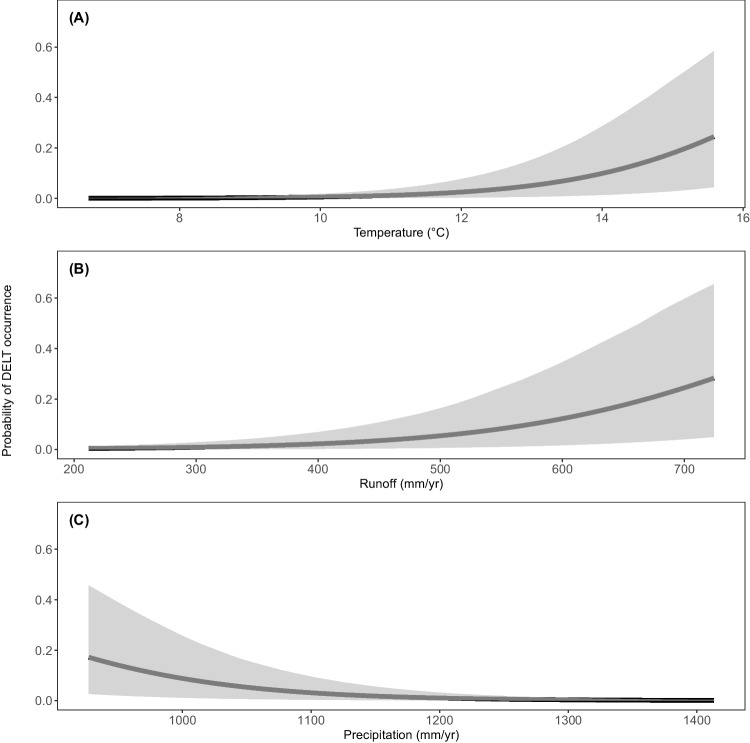
Predicted probability of DELT occurrence across all fish species sampled in the Chesapeake Bay watershed, USA (modeled using Eq. 2) as a function of air temperature (**A**), runoff (**B**), and precipitation (**C**). Solid line is posterior mean and shaded area is 90% credible region. All other predictor variables are held at average values found in this study

Of the 57 species, 28 (49%) had lower 90% CIs that did not include the value zero (Fig. 3), including four North American catfish (Ictaluridae) (Channel Catfish, Yellow Bullhead, Margined Madtom (*Noturus insignis*), and Brown Bullhead) and 7 sunfish (Centrarchidae) (Green Sunfish (*Lepomis cyanellus*), Bluespotted Sunfish (*Enneacanthus gloriosus*), Pumpkinseed (*Lepomis gibbosus*), Warmouth (*Lepomis gulosus*), Rock Bass, Largemouth Bass (*Micropterus salmoides*), and Smallmouth Bass). The collection agency-specific mean probability of DELT occurrence for the 7 collection agencies ranged from 0.0017 (90% CI = 0.0008, 0.0029) to 0.1913 (90% CI = 0.1370, 0.2502). The ecoregion-specific mean probability of DELT occurrence for the nine level 3 ecoregions that fish were collected from ranged from 0.012 to 0.031 for the Central Appalachians and Blue Ridge, respectively (mean = 0.020, 90% CI = 0.004, 0.053; Fig. A3).

### Landscape predictors of modeled DELT occurrence probability

There were a total of 46 landscape predictors included in the DELT model (in Eq. 2) that was fitted across all species. Of the 46 predictors, 33 were significant, 2 had a $pd>$ 0.90 and were potentially biologically significant, and 11 were not statistically significant (Fig. 4). Significant landscape predictor variables spanned a wide-range of categories describing upstream watershed characteristics; however, the three predictors with the largest effects were air temperature, runoff, and precipitation (Fig. 5). The modeled mean probability of DELT occurrence increased with increasing air temperature and runoff but declined with increasing precipitation. However, there is a large amount of uncertainty associated with the probability of DELT occurrence at higher temperatures and runoff values and at low levels of precipitation. For example, the 90% CI spanned two orders of magnitude at low mean temperatures ($\sim$7 $^{\circ }$C; 4.5$e^{-05}$ to 0.001) but spanned approximately one order of magnitude at higher mean temperatures ($\sim$16 $^{\circ }$C; 0.04 to 0.58). Similar patterns of the predicted magnitude of the probability of DELT occurrence and associated uncertainty were present for the effects of runoff and precipitation (Fig. 5).

Other significant predictors positively correlated with DELT occurrence probability across all fish species included landscape features related to altered land use, including housing and road densities (Fig. 4). Although significant, their effects on DELT were smaller than temperature and runoff and with less drastic increases in the probability of DELT with an increase of predictor values. However, uncertainty also increased with increases in these predictors (Fig. A4A and B). In contrast to housing and road densities, local septic density had a lower estimated effect but with higher uncertainty at increased levels (Fig. A4C). Geologic and soil characteristics, such as soil pH (Fig. A5A), clay content, sinkhole density, and rock calcium oxide (CaO) content, were also positively correlated with DELT occurrence (Fig. 4).

Agricultural predictors that were positively correlated with modeled DELT occurrence probability include conventional tillage practices and pesticide use in the upstream watershed. Pesticide use (kg/km$^2$) had a smaller effect on DELT occurrence than conventional tillage (Fig. A5B and C). As the percentage of land with conventional tillage increased, so did the uncertainty surrounding its effect on the probability of DELT occurrence. The probability of DELT occurrence minimally increased throughout a range of upstream pesticide use (Fig. A5C). Conversely, DELT probability of occurrence was negatively correlated with other agricultural predictors in the upstream watershed including, in ascending order of effect, agricultural total nitrogen reduction BMPs, pasture, land on which no tillage was practiced, irrigated agriculture, and cropland (Figs. 4 and A6).

Several landscape features representative of development and human alteration were also negatively correlated with DELT occurrence probability, including, in ascending order of effect size, major National Pollution Discharge Elimination System (NPDES; https://www.epa.gov/npdes/npdes-permit-basics) facility density, harvested forest, Facility Registry Service (FRS; https://www.epa.gov/frs) facility density, and extractive and impervious land in the upstream watershed (Figs. 4, A7A, and B). Hydrologic predictors were either positively or negatively related to DELT occurrence probability. Of the hydrologic predictors, stream order had the largest effect (positive) on DELT occurrence probability, followed by water table depth and NHD pipeline flowline (i.e., man-made structures of steel, concrete, or polymers that direct surface water flows), respectively (Fig. 4). Connector flowline density, base flow index, dam density, canal flowline density, basin slope, overland flow, and stream slope were all negatively correlated with DELT occurrence probability. Surprisingly, increased levels of conservation easements were related to increased DELT probability across all species (Fig. A7C). Within the landscape mosaic, landscape predictors of DELT occurrence probability were significant across all spatial scales and hierarchies of both the freshwater patch connectivity and the combined terrestrial and human patch contexts (Fig. A8).

**Fig. 6:**
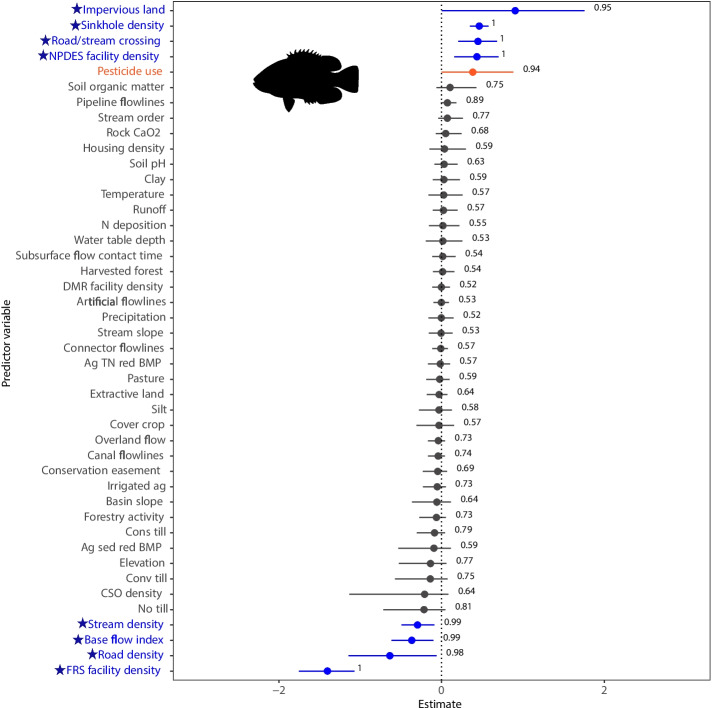
The effects of landscape predictors on the probability of DELT occurrence for Rock Bass (*Ambloplites rupestris*) in the Chesapeake Bay watershed, USA (modeled using Eq. 2, except without a species random effect). Estimated effects $(\beta )$ are shown on the x-axis as posterior means (circles) and 90% credible intervals (horizontal bars). Predictors marked with a star and highlighted in blue have 90% credible intervals that did not include the value zero, the predictor highlighted in orange (pesticide use) shows a high probability (*pd* > 0.90) that its estimated effect $(\beta )$ is in the direction (positive or negative) of the posterior mean, and predictors shown in grey have 90% credible intervals that did not include the value zero. The posterior probability for each predictor is displayed as a numerical value. Fish silhouette is from the R system rphylopic package (R Core Team, 2024; Gearty & Jones, 2023)

### Species-specific landscape predictors of modeled DELT occurrence probability

There were six species that met the criteria of having a naive DELT prevalence $\ge$ 5% and sample size > 100—the Channel Catfish (naive prevalence = 19%, *n* = 888), Golden Shiner (naive prevalence = 9%, *n* = 705), Rock Bass (naive prevalence = 8%, *n* = 7,518), Smallmouth Bass (naive prevalence = 7%, *n* = 10,557), Brown Bullhead (naive prevalence = 6%, *n* = 556), and Yellow Bullhead (naive prevalence = 5%, *n* = 2,993). The six species were collected from streams that varied in upstream watershed land use and cover (Fig. A9).

Modeled according to Eq. 2, except without a species random effect, the total number and type of significant landscape predictors of DELT occurrence probability varied by species. For both Brown Bullhead and Yellow Bullhead, no significant predictors were identified (Figs. A10 and A11). The DELT occurrence probability for both Smallmouth Bass and Channel Catfish was related to a single landscape predictor. The probability of DELT occurrence in Smallmouth Bass increased with increases in the percentage of harvested forest in the upstream watershed but with a large amount of uncertainty at high percentages of harvested forest (Figs. A12 and A13). Channel Catfish DELT occurrence probability decreased with increases of non-publicly owned treatment discharge monitoring report (DMR) location densities, with a large amount of uncertainty at lower densities (< 1 facility/km$^2$; Figs. A14 and A15). DMR locations are EPA-monitored industrial or commercial facilities that discharge wastewater directly into surface waters (Gordon et al., 2017). In comparison to all DELT collection streams, there was high agricultural land (median,  20%; Fig. A9) in the upstream watersheds of Channel Catfish collection streams, where industrial and commercial facilities are not common.

**Fig. 7:**
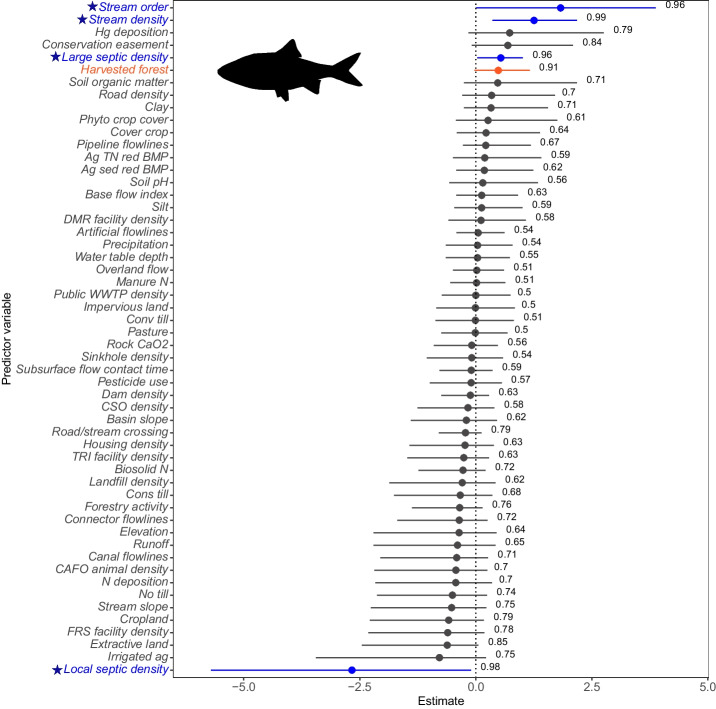
The effects of landscape predictors on the probability of DELT occurrence for Golden Shiner (*Notemigonus crysoleucas*) in the Chesapeake Bay watershed, USA (modeled using Eq. 2, except without a species random effect). Estimated effects $(\beta )$ are shown on the x-axis as posterior means (circles) and 90% credible intervals (horizontal bars). Predictors marked with a star and highlighted in blue have 90% credible intervals that did not include the value zero, the predictor highlighted in orange (Harvested forest) shows a high probability (*pd* > 0.90) that its estimated effect $(\beta )$ is in the direction (positive or negative) of the posterior mean, and predictors shown in grey have 90% credible intervals that overlap with zero. The posterior probability for each predictor is displayed as a numerical value. Fish silhouette is from the R system rphylopic package Fish silhouette is from the R system rphylopic package (R Core Team, 2024; Gearty & Jones, 2023)

The probability of Rock Bass DELT occurrence increased with increases in densities of impervious land, sinkholes, road/stream crossings, and NPDES facilities and with pesticide use and decreased with increases in stream density, base flow index, and densities of roads and FRS facilities (Fig. 6). Uncertainty in the predicted Rock Bass probability of DELT occurrence increased with increases in densities of impervious land, sinkholes, and road/stream crossings (Fig. A16A–C). In comparison, the predicted uncertainty for Rock Bass DELT probability was lower with increases in NPDES facility density and pesticide use (Fig. A16D-E) and with decreases in stream density, base flow index, and road and FRS facility densities (Fig. A17). Both FRS and NPDES are EPA tools for environmental regulation; however, each has a distinct role. The FRS encompasses a range of facilities across various media (e.g., air, water, and waste), whereas NPDES concentrates specifically on regulating water pollution (Gordon et al., 2017).

Golden Shiner DELT occurrence probability increased with increases in stream order and density, large septic facility density, and harvested forest (Fig. 7). Uncertainty in Golden Shiner DELT probabilities increased with increasing stream order, stream density, and large septic facility densities. However, in comparison, there was lower predicted uncertainty in Golden Shiner DELT probabilities with increases in harvested forest, although the effect was smaller (Fig. A18A-D). Golden Shiner DELT probability of occurrence decreased with increases in local (individual housing and facility) septic density and with higher predicted uncertainty in upstream watersheds with local septic densities of < 1 facility/km$^2$ (Fig. A18E).

## Discussion

### Observed DELT prevalence and modeled species-specific occurrence probability

External, visible abnormalities on fish have been monitored in many areas as an indicator of ecosystem health. Unlike more comprehensive indicators that include internal abnormalities, cellular and molecular biomarker responses, DELTs are a nonlethal method that can be conducted relatively quickly in the field during population or other monitoring activities. Although qualitative and typically measured at the community level (Sanders et al., 1999), DELTs are not diagnostic of specific health problems. However, DELTs are fairly simple to implement with minimal time and energy and can therefore generate large sample sizes (e.g., 577,266 individuals in the 12 years of this study).

Overall, the observed DELT prevalence in the Chesapeake Bay watershed was low (1.4%); prevalence of individual species ranged from 0 to 18.9%. The among-species variation may be attributed to a combination of species-specific traits, habitat characteristics, and land-use factors, but could also be due to the complexity of the dataset (refer to Limitations section) demonstrating that more focused research with standardized protocols could help explain these differences and identify drivers. The observed DELT prevalence documented in the literature ranges from 0 to 0.3% in high-quality streams, increasing to 5% in highly degraded streams (Bagley et al., 2025; Sanders et al., 1999; Simon & Emery, 1995). In this study, with respect to these designations, 53% of streams were categorized as high-quality and 12% as highly degraded, based solely on the observed DELT prevalence.

Comparisons of fish DELT across different regional watershed studies reveal varying prevalence rates, highlighting the potential influences of local environmental conditions and study methodologies. Overall DELT prevalence observed in the Chesapeake Bay watershed was 1.4% which is similar to observed prevalence of 2.6% in seven Ohio streams (Sanders et al., 1999) and 2.0% in four Illinois rivers (DeBoer et al., 2024). On the other hand, DELT prevalence in our study area was higher than the 0.17% in 150,000 individuals collected from 498 Alabama streams. Further, a Lake Erie study of 20 lacustrine and shoreline sites reported that 88% of sites had DELT present (Baumann et al., 2000) and a state-wide Alabama study of 498 streams reported 11% of sites had DELT present (Bagley et al., 2025), while in our study, DELT presence was observed in 53% of streams. Similar to the current study, in both Illinois and Alabama streams, the percentage of affected individuals varied among species (DeBoer et al., 2024; Bagley et al., 2025). Overall results varied by study, and these differences could be attributed to variations in the site land cover, species collected, study duration, size and spatial extent, or specific types of DELTs measured.

Notably, five of the six species exhibiting a DELT prevalence >5%—Channel Catfish, Rock Bass, Smallmouth Bass, Brown Bullhead, and Yellow Bullhead—were large (median common length = 23 cm) and long-lived (median max years = 18) species. In contrast, the Golden Shiner that also exhibited a DELT prevalence >5%, has a small size (common length = 14 cm) and short lifespan (max years = 9). Larger and longer-lived species may be more likely to have abnormalities due to extended exposure to contaminants (Volta et al., 2009; Pagano et al., 2021), opportunistic parasites, disease, and other environmental stressors. There may be other species-specific factors, such as habitat preference, feeding behavior and physiological factors, that influence responses to environmental stressors. Additionally, for sportfish such as bass and catfish, catch and release fishing which involves hooking stress and handling may also contribute to DELT occurrence (Cooke et al., 2002; Olsen et al., 2010). The species with higher DELT prevalence in this regional assessment have also exhibited increased DELT in more localized investigations (Sanders et al., 1999; Pinkney et al., 2004; DeBoer et al., 2024).

To our knowledge, there have been no studies focused on the health of Channel Catfish or Yellow Bullhead in the Chesapeake Bay watershed. Channel Catfish, Yellow Bullhead, and Brown Bullhead are bottom-dwelling species belonging to the North American catfish family Ictaluridae. Due to the development of skin and liver tumors in highly contaminated areas of concern, Brown Bullhead have been used extensively as an indicator species in the Great Lakes and Cape Cod regions and in tributaries of Chesapeake Bay (Rafferty et al., 2009; Baumann et al., 2008; Pinkney et al., 2009). The bottom-dwelling and bottom-feeding behavior exposes them to sediment contamination. Fournie et al. (1996) reported a higher prevalence of gross abnormalities in demersal compared to pelagic fishes. Notably, the prevalence was eight times higher at sites with sediment chemical contaminants such as metals, PAHs, and PCBs at concentrations above the ecological response criteria described by Long and Morgan (1990). Among the species examined in that study, the Brown Bullhead stood out with a particularly high prevalence of abnormalities, reaching 14.9% (Fournie et al., 1996). In three Potomac River tributaries sampled during 2009–2011 the percentage of Brown Bullhead with skin tumors ranged from 11 to 18%, with the highest percentages observed in urbanized watersheds (Pinkney et al., 2014). In the current analyses, no significant landscape predictors were identified for Brown or Yellow Bullhead.

Beginning in 2002, stream-reach intensive studies noted an increased prevalence of skin lesions in adult fish in the Susquehanna River, PA, and lesions and mortality events in the Potomac River, which led to concerns about the health of certain fish species in the Chesapeake Bay watershed (Blazer et al., 2010; Keplinger et al., 2022). The incidence of certain abnormalities, including melanistic areas on the body surface of Smallmouth Bass, varied from 2% in the Juniata River to 8% in the mainstem Susquehanna River, PA in 2012–2018 (Blazer et al., 2020). Prevalence of skin and lip tumors in Brown Bullhead collected in 2014–2016 ranged from 0 to 15% in Chesapeake Bay tributaries (Pinkney et al., 2019). In another study, DELT incidence was low (2.0%) at multiple sites in Illinois rivers with Channel Catfish (7.9%) and Smallmouth Bass (4.9%) among the top species exhibiting DELT (DeBoer et al., 2024). Smallmouth Bass have also been used in the Great Lakes, Chesapeake Bay watershed, and numerous other watersheds as an indicator species (Blazer et al., 2010; Starliper et al., 2013; Walsh et al., 2022; Dieterman et al., 2019). Recent research suggests that there was significant annual variation in different types of abnormalities (Keplinger et al., 2022), indicating that seasonal sampling can also influence DELT patterns depending on when fish are collected (Jensen, 2023). For example, melanistic areas, or “blotchy bass,” is caused by a virus and has also been observed in Smallmouth Bass, with a higher prevalence in cooler temperatures (Schall et al., 2025).

In this study, most abnormalities were identified as parasites (60.5%), followed by lesions (23.0%), erosions (13.1%), deformities (1.9%), tumors (0.8%), and other (0.9%). Parasites have been suggested as indicators of aquatic ecosystem health (Lafferty, 1997; Marcogliese, 2005; Sures et al., 2017); however, the relationship among parasites, their hosts, and the environment is complex. Fish parasites have both direct and indirect life cycles, and factors related to parasite proliferation, reproduction, transmission, and infectivity often need to be considered in addition to environmental stressors that influence the host immune response. Some parasites, such as leeches (Sket & Trontelj, 2008) and monogenetic trematodes (Cribb et al., 2002), have direct life cycles. Conversely, other parasites, such as digenetic trematodes, which cause abnormalities such as black spot and raised white to yellow cysts, or myxozoans, which cause small white cysts on gills and body surface, have indirect life cycles that include snails, annelids, and other benthic invertebrates as intermediate hosts, as well as free-swimming infective stages (Cribb et al., 2002; Lisnerová et al., 2020). These free-living stages are susceptible to environmental stressors; hence, the presence of certain parasites may indicate a “healthier watershed,” while other parasite taxa may be indicative of more contaminated or altered habitats (Blanar et al., 2009; Vidal-Martinez et al., 2010; Sures et al., 2017). Consequently, it would be informative to identify the types of parasites, when possible, during DELT surveys.

### Landscape predictors and best management practices

The DELT data were modeled with numerous landscape variables to determine potential drivers of fish health on a regional scale. Across all species, air temperature and runoff in the upstream watershed had the largest effect sizes and were positively correlated with DELT occurrence probability, while precipitation in the upstream watershed had the strongest negative effect, despite the large amount of uncertainty. While elevated water temperatures can promote proliferation of certain parasites and pathogens, for others, increased water temperature can decrease or have no effect on prevalence (Marcogliese, 2008; Karvonen et al., 2010; Marcos-López et al., 2010). Further, a global meta-analysis explored the effect of season on parasite infections and found that while some parasite families showed seasonal variation, others did not (Poulin, 2020). This demonstrates the potential utility of additional temporal assessments of DELT broadly across the Chesapeake Bay watershed.

The lack of significant predictors for members of the North American catfish family (Channel Catfish, Yellow Bullheads, and Brown Bullheads) may be due to their shared benthic lifestyles and tolerance for varied environmental conditions (Haubrock et al., 2021; Fish & Game, 2024). In this study, the three species were collected in areas with similar median percentages of development (roughly 10%). Rock Bass, in comparison, were more sensitive to anthropogenic stressors, with their DELT occurrence probability linked to impervious land, sinkhole density, road/stream crossings, NPDES facility density, and pesticide use, particularly in more agriculturally impacted but less developed streams, in comparison to all DELT collection streams. These stressors can contribute to flash flooding, habitat loss, warmer water temperatures, and pollution, which can negatively impact fish health (Webster, 2020; Blazer et al., 2013; Matsche et al., 2023).

Unlike Rock Bass, Golden Shiner DELT occurrence probability was positively related to increased stream density and stream size (stream order). Differences in collection streams may play a role: Golden Shiners were collected from more developed, less agricultural or forested streams compared to Rock Bass (Fig. A9), where pollutant loads in larger streams may have a greater effect on fish health. Furthermore, Golden Shiners were collected from more densely populated areas (where large septic densities correlated with DELT) than from rural ones (where local septic density correlated inversely with DELT occurrence probability).

The only significant landscape predictor for DELT in Smallmouth Bass identified in the current study was harvested forest. Effects of forest harvest on the aquatic ecosystems are variable and depend on the type of harvest, as well as stream size, stream gradient, habitat, and underlying environmental variables (Richardson & Béraud, 2014). Increased flow, stream temperature, suspended sediment, and nutrients have been reported during and after harvest (Shah et al., 2022). Forest harvest has also been shown to increase mercury concentrations in fish collected from affected watersheds (Wu et al., 2018). A recent Chesapeake Bay watershed study examined mercury fillet concentrations in 32 fish species. Substantial spatial variation was observed; however, popular sportfish including Walleye (*Sander vitreus*), Striped Bass (*Morone saxatilis*), and Largemouth and Smallmouth Bass had the highest mean concentrations (Willacker et al., 2020). Biological data from that study also showed total mercury concentrations in Smallmouth Bass correlated with an increase in external and internal visible abnormalities and tissue parasite burdens (Blazer et al., 2023).

Several landscape predictors showed unexpected correlations with DELT occurrence probability (Fig. 4), including densities of extractive land (e.g., mining, drilling), impervious surfaces, industrial and discharge facilities, and land under conservation easements. Extractive land often exists in remote areas, while impervious surfaces, in this study, encompass a range of developed landscape intensities, from open spaces to highly altered urban areas. Conservation easement land, as reported by the USDA NRCS (https://www.nrcs.usda.gov/programs-initiatives/ale-agricultural-land-easements), includes active agricultural areas like cropland, rangeland, and pastureland (Wieczorek et al., 2018). These easements aim to prevent conversion of active agricultural land to non-agricultural uses, and continuing agricultural practices could potentially influence surrounding aquatic ecosystems.

The DELT occurrence probability across all species (modeled using Eq. 1) decreased with increases in pastureland, irrigated agriculture, and cropland within the upstream watershed, indicating fish in these more rural areas may be less impacted than those in highly developed areas. Conversely, conventional tillage practice was positively correlated with the overall DELT occurrence probability across all species. Conventional tillage can increase sediment loads, contributing to biological impairment in Chesapeake Bay watershed streams (Noe et al., 2020; Maloney et al., 2021). In contrast, the DELT occurrence probability decreased with increasing no-tillage practices, potentially due to the reduced sediment load to streams that are associated with these practices.

Nutrient and sediment BMPs are mostly intended to reduce nutrients and sediments from entering aquatic ecosystems (Risal & Parajuli, 2022), thus reducing loads to the Chesapeake Bay. Any improvements in fish health would be considered a co-benefit of these BMPs. In our study, streams with available DELT data had upstream watersheds that included an average of 16% agriculture, and we observed a potential decrease in DELT probability across species with increased total nitrogen reduction due to BMP implementation. The total nitrogen reduction due to BMP implementation at DELT collection streams was an estimated value for the year 2012, which was based on modeling of the expected effects of the implemented BMPs in the upstream watershed of each site on reductions in downstream loading of nitrogen. This estimate represents the combined nitrogen losses from runoff, water and wind erosion, tile drainage, percolation, and subsurface and quick return flow pathways (Wieczorek et al., 2018) but does not include nitrogen reduction from urban management controls like stormwater and infiltration basins. Nutrient enrichment has been linked with increases in fish skin lesions and high helminth and myxozoan parasites loads (McKenzie & Townsend, 2007; Pulkkinen & Taskinen, 2024). Excessive nutrients can increase stream eutrophication that has the potential to result in larger populations of fish parasite vectors (Budria, 2017; Marcogliese & Cone, 2021). However, in a separate study, BMPs did not affect fish DELT in small Chesapeake Bay watershed tributaries originating in upstream watersheds primarily composed of mixed forest and pastureland, and it was suggested that agricultural pollutants might cause more internal endocrine effects rather than visible DELT (Jensen, 2023).

Further research would be needed to evaluate the role of BMPs in mixed land-use streams within the Chesapeake Bay watershed because it remains unclear how well BMPs largely intended to address nitrogen, phosphorus, and sediment, address stream health issues arising from a mixture of urban and agricultural land. Ongoing studies are aimed at developing a holistic understanding of Chesapeake Bay watershed stream ecosystems by considering diverse stressors and BMP effectiveness, which could guide future efforts to improve water quality and ecosystem health (Noe et al., 2024). Future research could investigate the Chesapeake Bay watershed’s landscape mosaic and how changes in freshwater connectivity and terrestrial landscapes influence DELT over time. This could include examining factors like precipitation and patch connectivity, as well as exploring how temperature and land-use changes impact fish health in the broader terrestrial and human landscape contexts.

## Limitations

Our study highlights the value, but also highlights limitations, of analyzing a large regional DELT dataset with multiple collection agencies. The lack of commonality in documenting abnormalities among collection agencies presents difficulties in harmonizing observations across measurement protocols to a meaningful categorization of DELT types (e.g., consistent definitions of “lesion” or “tumor” across all agencies). For instance, cysts and white spots were included in most Chesapeake Bay watershed surveys, but not all surveys included leeches and anchor worms. Most agencies did not include gill abnormalities but some did, which complicated efforts to accurately evaluate and analyze specific categories of abnormalities. Therefore, our analyses focused on the presence of any abnormality (DELT occurrence) as a comprehensive indicator of fish health. We acknowledge that this approach means an observation such as a black spot (not likely affecting the health of the fish) is not differentiated from a large ulceration of the skin or emaciation (more likely to affect health and survival). Resource managers can improve data quality and usability by focusing on clear and detailed descriptions of DELT anomalies and categories supported by standardized training to fish health observers. This would require field crews to be consistent in what they are documenting and how the DELT anomalies are defined.

Differences in sampling seasons across agencies may also skew the overall DELT observations. In particular seasons, species-specific life history events such as spawning can increase DELT occurrences in the field. Most fish in this study were collected in the summer or fall. Although most collections commenced on June 1st, coinciding with spawning for some species (e.g., catfish, sunfish), this collection period was designed to minimize, though may not eliminate, all spawning effects on DELT observations. Nevertheless, we acknowledge that seasonal variation in the observations of species-specific DELT and in overall DELT (e.g., from increased angler handling in summer months) may still exist. Differences in dominant sampling seasons across agencies (e.g., some agencies collected more during late fall to winter) have the potential to skew the overall DELT observations in this study. The potential for temporal variations in DELT across species highlights the need to account for seasonal influences in future studies, particularly those interested in assessing specific relationships with changes in landscape drivers.

The landscape predictor analyses demonstrated a high level of uncertainty, which is likely due to the low prevalence of DELT and the use of mean annual measurements for some landscape variables. For many of the variables such as temperature, pesticide use and runoff, the annual mean is likely less meaningful in relation to DELT, than upstream watershed-specific seasonal variations. However, because this study used a spatial framework focused on geographic variation in upstream watersheds and not on temporal changes, we relied on annual means due to data availability and study design. Future research incorporating a temporal approach could explore the use of seasonal or daily averages to better capture environmental dynamics and potentially reduce uncertainty in predictor-response relationships. Beyond that, a key limitation was the correlational nature of the study, which makes it impossible to infer causation or determine possible mechanisms or risk factors, in term of landscape predictors. Some of the observed correlations may be spurious or represent proxies for other drivers in the system affecting DELT occurrence, for instance, continuing agricultural practices on conservation easement land.

## Conclusion

The study presents a detailed assessment of DELT occurrence on a regional level within the Chesapeake Bay watershed. Observations of DELT are a broad measure of fish health but are a relatively coarse indicator that cannot identify specific causes of disease. Notably, the low overall DELT occurrence probability in the Chesapeake Bay watershed indicates that DELT are rarely observed. The analysis identified specific landscape predictors that warrant further investigation and species that may serve as regional indicators for environmental effects on fish health in future studies, including Rock Bass, Golden Shiner, Smallmouth Bass, Channel Catfish, Brown Bullhead, and Yellow Bullhead. Numerous factors are important to consider in understanding “causes” or risk factors of external abnormalities. Fish traits such as life history, habitat usage, susceptibility to specific pathogens, physiological sensitivity to contaminants, and other stressors are important. Future research could focus on more localized studies or on species-specific responses to stressors through longitudinal studies that incorporate life stages and health indicators, further contributing to the conservation of freshwater ecosystems and sustainable fish health management. This initial landscape-level analysis serves as a crucial first step in directing future research towards potentially influential environmental drivers of fish health.

## Appendix

Supplementary information is included in the Appendix and contains additional DELT category, DELT agency crosswalk, landscape predictor definition, and observed DELT occurrence tables and also contains additional figures including maps, land-use plots, modeled land-use effects across all species, landscape mosaic chart, and species-specific DELT models.

## Disclosure

Any use of trade, firm, or product names is for descriptive purposes only and does not imply endorsement by the U.S. Government.

## Supplementary Information

Below is the link to the electronic supplementary material.

Supplementary file 1 (pdf 3703 KB)

## Data Availability

Sampling agencies included the U.S. Environmental Protection Agency (USEPA) National Rivers and Streams Assessment (NRSA) (USEPA, 2019b, a), Virginia Interactive Stream Assessment (INSTAR) (Virginia Commonwealth University, 2023), Maryland Department of Natural Resources (MDDNR) (Stranko et al., 2007), Montgomery County Department of Environmental Protection (MCDEP, 2023), Pennsylvania Department of Environmental Protection (PADEP) (Lookenbill & Whiteash, 2021), Virginia Department of Environmental Quality (VADEQ) (https://www.deq.virginia.gov/our-programs/water/water-quality/monitoring/probabilistic-monitoring), and Virginia Department of Wildlife Resources (VDWR) (Virginia Department of Wildlife Resources, 2020). USEPA fish community data are available online at USEPA (2019b). Requests for all other fish community data can be made to state fish and wildlife agencies, which we obtained under data sharing agreements. The code for model fitting and posterior inference is available at https://doi.org/10.5066/P1NJV3HQ.

## References

[CR1] Adams, S. (2002). *Biological indicators of aquatic ecosystem stress* (pp. 631–644). Oak Ridge National Laboratory, New York: American Fisheries Society. Environmental Sciences Division.

[CR2] Bagley, J. C., Phillips, A. K., Buchanon, S., O’Neil, P. E., & Huff, E. S. (2025). Incidence and effects of anomalies and hybridization on Alabama freshwater fish index of biotic integrity results. *Environmental Monitoring and Assessment,**197*, 1–16. 10.1007/s10661-024-13512-2 39661207

[CR3] Baumann, P., Cairns, V., Kurey, B., Lambert, L., Smith, I., & Thoma, R. (2000).* Lake Erie Lakewide Management Plan (LaMP)*. Technical Report Series. Lake Erie LaMP Technical Report No. 6) https://nepis.epa.gov/Exe/ZyPDF.cgi/P1012I1O.PDF?Dockey=P1012I1O.PDF.

[CR4] Baumann, P. C., LeBlanc, D. R., Blazer, V., Meier, J. R., Hurley, S. T., & Kiryu, Y. (2008). *Prevalence of tumors in brown bullhead from three lakes in Southeastern Massachusetts, 2002*. US Geological Survey: Technical report.

[CR5] Becker, R. A., Wilks, A. R., Brownrigg, R., Minka, T. P., & Deckmyn, A. (2023). *maps: draw geographical maps*. https://CRAN.R-project.org/package=maps.

[CR6] Biggs, R., Carpenter, S. R., & Brock, W. A. (2009). Spurious certainty: how ignoring measurement error and environmental heterogeneity may contribute to environmental controversies. *BioScience,**59*, 65–76.

[CR7] Blanar, C. A., Munkittrick, K. R., Houlahan, J., MacLatchy, D. L., & Marcogliese, D. J. (2009). Pollution and parasitism in aquatic animals: a meta-analysis of effect size. *Aquatic Toxicology,**93*, 18–28. 19349083 10.1016/j.aquatox.2009.03.002

[CR8] Blazer, V., Iwanowicz, L. R., Starliper, C. E., Iwanowicz, D., Barbash, P., Hedrick, J., Reeser, S., Mullican, J., Zaugg, S., Burkhardt, M., et al. (2010). Mortality of centrarchid fishes in the Potomac drainage: survey results and overview of potential contributing factors. *Journal of Aquatic Animal Health,**22*, 190–218. 21192549 10.1577/H10-002.1

[CR9] Blazer, V., Mazik, P. M., Iwanowicz, L. R., Braham, R. P., Hahn, C. M., Walsh, H. L., & Sperry, A. J. (2014). *Assessment of the fish tumor beneficial use impairment in brown bullhead (Ameiurus nebulosus) at selected Great Lakes Areas of Concern*. US Geological Survey: Technical report.

[CR10] Blazer, V. S., Pinkney, A. E., Jenkins, J. A., Iwanowicz, L. R., Minkkinen, S., Draugelis-Dale, R. O., & Uphoff, J. H. (2013). Reproductive health of yellow perch Perca flavescens in selected tributaries of the Chesapeake Bay. *Science of the Total Environment,**447*, 198–209. 23384644 10.1016/j.scitotenv.2012.12.088

[CR11] Blazer, V. S., Walsh, H. L., Sperry, A. J., Raines, B., Willacker, J. J., & Eagles-Smith, C. A. (2023). A multi-level assessment of biological effects associated with mercury concentrations in smallmouth bass. *Micropterus dolomieu. Environmental Pollution,**329*, 121688. 37088253 10.1016/j.envpol.2023.121688

[CR12] Blazer, V. S., Young, K. T., Smith, G. D., Sperry, A. J., & Iwanowicz, L. R. (2020). Hyperpigmented melanistic skin lesions of smallmouth bass Micropterus dolomieu from the Chesapeake Bay watershed. *Diseases of Aquatic Organisms,**139*, 199–212. 32495746 10.3354/dao03480

[CR13] Blodgett, D., & Johnson, M. (2023). *NHDPlusTools: tools for accessing and working with the NHDPlus*. Reston, VA. 10.5066/P97AS8JD.

[CR14] Budria, A. (2017). Beyond troubled waters: the influence of eutrophication on host-parasite interactions. *Functional Ecology,**31*, 1348–1358.

[CR15] Carpenter, B., Gelman, A., Hoffman, M. D., Lee, D., Goodrich, B., Betancourt, M., Brubaker, M. A., Guo, J., Li, P., & Riddell, A. (2017). Stan: a probabilistic programming language. *Journal of Statistical Software,**76*. 10.18637/jss.v076.i01 PMC9788645 36568334

[CR16] Chesapeake Bay Program. (2022). *Chesapeake Bay Program Quick Reference Guide for Best Management Practices (BMPs): nonpoint source BMPs to reduce nitrogen, phosphorus and sediment loads to the Chesapeake Bay and its local waters*. Chesapeake Bay Program, second edition. https://www.chesapeakebay.net/files/documents/BMP-Guide_Full.pdf.

[CR17] Chesapeake Bay Program. (2023). *Chesapeake Bay Land Use and Land Cover (LULC) Database 2022 Edition*. 10.5066/P981GV1L.

[CR18] Chesapeake Bay Program. (2025a). *The Chesapeake Bay Watershed*. https://www.chesapeakebay.net/discover/watershed. Accessed 19-May-2025

[CR19] Chesapeake Bay Program. (2025b). *Chesapeake Bay Watershed Agreement*. https://www.chesapeakebay.net/what/what-guides-us/watershed-agreement. Accessed 25-June-2025

[CR20] Chesapeake Bay Program. (2025c). *Planning for 2025 and Beyond*. https://www.chesapeakebay.net/what/what-guides-us/planning-for-2025-and-beyond. Accessed 25-June-2025

[CR21] Cooke, S. J., Schreer, J. F., Wahl, D. H., & Philipp, D. P. (2002). Physiological impacts of catch-and-release angling practices on largemouth bass and smallmouth bass. In *American fisheries society symposium*, (pp. 489–512). Citeseer.

[CR22] Cribb, T. H., Chisholm, L. A., & Bray, R. A. (2002). Diversity in the Monogenea and Digenea: does lifestyle matter? *International Journal for Parasitology,**32*, 321–328. 11835972 10.1016/s0020-7519(01)00333-2

[CR23] DeBoer, J. A., Whitten Harris, A. L., Kuppek, V. E., Stafford, P. A., & Lamer, J. T. (2024). *External abnormalities in fish assemblages from four Illinois rivers*. INHS Technical Report 2024, (vol. 04).

[CR24] Dieterman, D. J., Hoxmeier, R. J. H., & Krumm, E. J. (2019). Associations between biotic integrity and sport fish populations in upper Midwest, USA rivers, with emphasis on Smallmouth Bass. *Environmental Management,**63*, 732–746. 30923958 10.1007/s00267-019-01156-9

[CR25] U.S. Environmental Protection Agency. (2010). *Level III and IV Ecoregions of the Continental United States*. U.S. EPA Office of Research & Development (ORD) - National Health and Environmental Effects Research Laboratory (NHEERL). https://www.epa.gov/eco-research/level-iii-and-iv-ecoregions-continental-united-states.

[CR26] Fish, N. H., & Game. (2024). *Yellow Bullhead*. https://www.wildlife.nh.gov/fishing-new-hampshire/fish-species-nh/yellow-bullhead.

[CR27] Fournie, J. W., Summers, J. K., & Weisberg, S. B. (1996). Prevalence of gross pathological abnormalities in estuarine fishes. *Transactions of the American Fisheries Society,**125*, 581–590.

[CR28] Gabry, J., & Češnovar, R. (2022). *CmdStanr: R Interface to ’CmdStan’*.

[CR29] Gearty, W., & Jones, L. A. (2023). rphylopic: An R package for fetching, transforming, and visualising PhyloPic silhouettes. *Methods in Ecology and Evolution,**14*, 2700–2708.

[CR30] Gelman, A., & Hill, J. (2006). *Data analysis using regression and multilevel/hierarchical models*. Cambridge University Press.

[CR31] Gordon, S., Jones, D., Williams, B., & Wright, C (2017). *Potential contaminant sources and other landscape variables summarized for NHDPlus Version 2.1 catchments within the Chesapeake Bay Watershed *(ver. 2.0, June 2021). 10.5066/F7SQ8ZB3.

[CR32] Gordon, S., Wagner, T., Smalling, K., & Devereux, O. (2023). Estrogenic activity response to best management practice implementation in agricultural watersheds in the Chesapeake Bay watershed. *Journal of Environmental Management,**326*, 116734. 36384057 10.1016/j.jenvman.2022.116734

[CR33] Grames, E. M., & Forister, M. L. (2024). Sparse modeling for climate variable selection across trophic levels. *Ecology*, e4231. 10.1002/ecy.4231 38290162

[CR34] Guardian, M. G. E., He, P., Bermudez, A., Duan, S., Kaushal, S. S., Rosenfeldt, E., & Aga, D. S. (2021). Optimized suspect screening approach for a comprehensive assessment of the impact of best management practices in reducing micropollutants transport in the Potomac River watershed. *Water Research X,**11*, 100088. 33598649 10.1016/j.wroa.2021.100088 PMC7868815

[CR35] Harris, J. H. (1995). The use of fish in ecological assessments. *Australian Journal of Ecology,**20*, 65–80. https://onlinelibrary.wiley.com/doi/abs/10.1111/j.1442-9993.1995.tb00523.x.

[CR36] Haubrock, P. J., Copp, G. H., Johović, I., Balzani, P., Inghilesi, A. F., Nocita, A., & Tricarico, E. (2021). North American channel catfish, Ictalurus punctatus: a neglected but potentially invasive freshwater fish species? *Biological Invasions,**23*, 1563–1576.

[CR37] Herlihy, A. T., & Sifneos, J. C. (2008). Developing nutrient criteria and classification schemes for wadeable streams in the conterminous US. *Journal of the North American Benthological Society,**27*, 932–948.

[CR38] Higgins, J. V., Bryer, M. T., Khoury, M. L., & Fitzhugh, T. W. (2005). A freshwater classification approach for biodiversity conservation planning. *Conservation Biology,**19*, 432–445.

[CR39] Jensen, J. B. (2023). *A Comparison of Fish Health Indices Applied to Freshwater Species of the Chesapeake Bay Watershed*. West Virginia University.

[CR40] Jones, J., Doctor, D., Wood, N., Falgout, J., & Rapstine, N. (2021). *Closed depression density in karst regions of the conterminous United States: features and grid data*. 10.5066/P9EV2I12.

[CR41] Karr, J. R. (1981). Assessment of biotic integrity using fish communities. *Fisheries,**6*, 21–27.

[CR42] Karvonen, A., Halonen, H., & Seppälä, O. (2010). Priming of host resistance to protect cultured rainbow trout Oncorhynchus mykiss against eye flukes and parasite-induced cataracts. *Journal of Fish Biology,**76*, 1508–1515. 20537029 10.1111/j.1095-8649.2010.02597.x

[CR43] Keplinger, B., Hedrick, J., & Blazer, V. S. (2022). Temporal trends in macroscopic indicators of fish health in the South Branch Potomac River. *North American Journal of Fisheries Management,**42*, 277–294.

[CR44] Kroon, F., Streten, C., & Harries, S. (2017). A protocol for identifying suitable biomarkers to assess fish health: A systematic review. *PloS One,**12*, e0174762. 28403149 10.1371/journal.pone.0174762 PMC5389625

[CR45] Lafferty, K. (1997). Environmental parasitology: what can parasites tell us about human impacts on the environment? *Parasitology Today,**13*, 251–255. 15275061 10.1016/s0169-4758(97)01072-7

[CR46] Lisnerová, M., Fiala, I., Cantatore, D., Irigoitia, M., Timi, J., Pecková, H., Bartošová-Sojková, P., Sandoval, C. M., Luer, C., Morris, J., et al. (2020). Mechanisms and drivers for the establishment of life cycle complexity in myxozoan parasites. *Biology,**9*, 10. 31906274 10.3390/biology9010010 PMC7168919

[CR47] Long, E. R., & Morgan, L. G. (1990). *The potential for biological effects of sediment-sorbed contaminants tested in the National Status and Trends Program*. National Oceanic and Atmospheric Administration: US Department of Commerce.

[CR48] Lookenbill, M., & Whiteash, R. (2021). *Water Quality Monitoring Protocols for Streams and Rivers*. Technical report, Pennsylvania Department of Environmental Protection, Harrisburg, Pennsylvania. https://files.dep.state.pa.us/water/Drinking%20Water%20and%20Facility%20Regulation/WaterQualityPortalFiles/Technical%20Documentation/MONITORING_BOOK.pdf.

[CR49] Lu, Z., & Lou, W. (2022). Bayesian approaches to variable selection: a comparative study from practical perspectives. *The International Journal of Biostatistics,**18*, 83–108. 10.1515/ijb-2020-0130 33761580

[CR50] Makowski, D., Ben-Shachar, M. S., Chen, S. A., & Lüdecke, D. (2019). Indices of effect existence and significance in the Bayesian framework. *Frontiers in Psychology,**10*, 2767. 31920819 10.3389/fpsyg.2019.02767 PMC6914840

[CR51] Maloney, K. O., Carlisle, D. M., Buchanan, C., Rapp, J. L., Austin, S. H., Cashman, M. J., & Young, J. A. (2021). Linking altered flow regimes to biological condition: an example using benthic macroinvertebrates in small streams of the Chesapeake Bay Watershed. *Environmental Management,**67*, 1171–1185. 33710388 10.1007/s00267-021-01450-5 PMC8106597

[CR52] Marcogliese, D. J. (2005). Parasites of the superorganism: are they indicators of ecosystem health? *International Journal for Parasitology,**35*, 705–716. 15925594 10.1016/j.ijpara.2005.01.015

[CR53] Marcogliese, D. (2008). The impact of climate change on the parasites and infectious diseases of aquatic animals. *Revue Scientifique et Technique,**27*, 467–484. 18819673

[CR54] Marcogliese, D. J., & Cone, D. K. (2021). Myxozoan communities in two cyprinid fishes from mesotrophic and eutrophic rivers. *The Journal of Parasitology,**107*, 39–47. 33535231 10.1645/20-76

[CR55] Marcos-López, M., Gale, P., Oidtmann, B., & Peeler, E. (2010). Assessing the impact of climate change on disease emergence in freshwater fish in the United Kingdom. *Transboundary and Emerging Diseases,**57*, 293–304. 20561287 10.1111/j.1865-1682.2010.01150.x

[CR56] Matsche, M. A., Blazer, V. S., & Pulster, E. L. (2023). White perch health relative to urbanization and habitat degradation in Chesapeake Bay tributaries. I. Biliary neoplasms and hepatic lesions. *Diseases of Aquatic Organisms,**154*, 85–105. 37410430 10.3354/dao03733

[CR57] MCDEP. (2023). *Montgomery County Department of Environmental Protection- Fish of Montgomery County*. https://www.montgomerycountymd.gov/. Accessed 11-Nov-2023

[CR58] McKay, L., Bondelid, T., Dewald, T., Johnston, J., Moore, R., & Rea, A. (2012). *NHDPlusV2 User Guide*. https://www.epa.gov/waterdata/nhdplus-national-hydrography-dataset-plus.

[CR59] McKenzie, V. J., & Townsend, A. R. (2007). Parasitic and infectious disease responses to changing global nutrient cycles. *EcoHealth,**4*, 384–396.

[CR60] Noe, G., Angermeier, P. L., Barber, L. B., Buckwalter, J., Cashman, M. J., Devereux, O., Doody, T. R., Entrekin, S., Fanelli, R. M., Hitt, N., et al. (2024). *Connecting conservation practices to local stream health in the Chesapeake Bay watershed*. US Geological Survey: Technical report.

[CR61] Noe, G. B., Cashman, M. J., Skalak, K., Gellis, A., Hopkins, K. G., Moyer, D., Webber, J., Benthem, A., Maloney, K., Brakebill, J., et al. (2020). Sediment dynamics and implications for management: State of the science from long-term research in the Chesapeake Bay watershed, USA. *Wiley Interdisciplinary Reviews: Water,**7*, e1454.

[CR62] Norton, S. B., Cormier, S. M., Suter, G. W., Subramanian, B., Lin, E., Altfater, D., & Counts, B. (2002). Determining probable causes of ecological impairment in the Little Scioto River, Ohio, USA: Part 1. Listing candidate causes and analyzing evidence. *Environmental Toxicology and Chemistry: An International Journal,**21*, 1112–1124. 12069294

[CR63] Olsen, R. E., Næsje, T. F., Poppe, T., Sneddon, L., & Webb, J. (2010). *Risk assessment of catch and release*.

[CR64] Pagano, J. J., Garner, A. J., Weidel, B., McGoldrick, D. J., Walsh, M., & Holsen, T. M. (2021). Legacy contaminant-stable isotope-age relationships in Lake Ontario year-class Alewife (Alosa pseudoharengus). *Journal of Great Lakes Research,**47*, 1086–1096.

[CR65] Piironen, J., & Vehtari, A. (2017). *Sparsity information and regularization in the horseshoe and other shrinkage priors*. Project Euclid.

[CR66] Pinkney, A. E., Harshbarger, J. C., May, E. B., & Reichert, W. L. (2004). Tumor prevalence and biomarkers of exposure and response in brown bullhead (Ameiurus nebulosus) from the Anacostia River, Washington, DC and Tuckahoe River, Maryland, USA. *Environmental Toxicology and Chemistry,**23*, 638–647. 15285357 10.1897/03-77

[CR67] Pinkney, A. E., Harshbarger, J. C., & Rutter, M. A. (2009). Tumors in brown bullheads in the Chesapeake Bay watershed: analysis of survey data from 1992 through 2006. *Journal of Aquatic Animal Health,**21*, 71–81. 10.1577/H08-037.1 19873828

[CR68] Pinkney, A., Harshbarger, J., & Rutter, M. (2014). Temporal and spatial patterns in tumour prevalence in brown bullhead Ameiurus nebulosus (Lesueur) in the tidal Potomac River watershed (USA). *Journal of Fish Diseases,**37*, 863–876. 24974857 10.1111/jfd.12271

[CR69] Pinkney, A. E., Harshbarger, J. C., Rutter, M. A., & Sakaris, P. C. (2019). Trends in liver and skin tumor prevalence in brown bullhead (Ameiurus nebulosus) from the Anacostia River, Washington, DC, and nearby waters. *Toxicologic Pathology,**47*, 174–189. 30798780 10.1177/0192623318823150

[CR70] Pinna, M., Zangaro, F., Saccomanno, B., Scalone, C., Bozzeda, F., Fanini,L., & Specchia, V. (2023). An Overview of Ecological Indicators of Fish to Evaluate the Anthropogenic Pressures in Aquatic Ecosystems: From Traditional to Innovative DNA-Based Approaches. *Water**15*. https://www.mdpi.com/2073-4441/15/5/949.

[CR71] Poiani, K. A., Richter, B. D., Anderson, M. G., & Richter, H. E. (2000). Biodiversity conservation at multiple scales: functional sites, landscapes, and networks. *BioScience,**50*, 133–146.

[CR72] Poulin, R. (2020). Meta-analysis of seasonal dynamics of parasite infections in aquatic ecosystems. *International Journal for Parasitology,**50*, 501–510. 32380095 10.1016/j.ijpara.2020.03.006

[CR73] Pulkkinen, K., & Taskinen, J. (2024). Nutrient enrichment increases virulence in an opportunistic environmental pathogen, with greater effect at low bacterial doses. *FEMS Microbiology Ecology,**100*, fiae013. 38305097 10.1093/femsec/fiae013 PMC10959552

[CR74] R Core Team. (2024). *R: A Language and Environment for Statistical Computing*. R Foundation for Statistical Computing, Vienna, Austria. https://www.R-project.org/.

[CR75] Rafferty, S. D., Blazer, V. S., Pinkney, A. E., Grazio, J. L., Obert, E. C., & Boughton, L. (2009). A historical perspective on the “fish tumors or other deformities’’ beneficial use impairment at Great Lakes Areas of Concern. *Journal of Great Lakes Research,**35*, 496–506.

[CR76] Richardson, J. S., & Béraud, S. (2014). Effects of riparian forest harvest on streams: a meta-analysis. *Journal of Applied Ecology,**51*, 1712–1721.

[CR77] Richkus, J., Wainger, L. A., & Barber, M. C. (2016). Pathogen reduction co-benefits of nutrient best management practices. *PeerJ,**4*, e2713. 27904807 10.7717/peerj.2713 PMC5126620

[CR78] Risal, A., & Parajuli, P. B. (2022). Evaluation of the impact of best management practices on streamflow, sediment and nutrient yield at field and watershed scales. *Water Resources Management,**36*, 1093–1105.

[CR79] Sanders, R. E., Miltner, R. J., Yoder, C. O., & Rankin, E. T. (1999). The use of external deformities, erosion, lesions, and tumors (DELT anomalies) in fish assemblages for characterizing aquatic resources: a case study of seven Ohio streams. In *Assessing the sustainability and biological integrity of water resources using fish communities*, pp. 225–246. CRC press.

[CR80] Schall, M. K., Smith, G. D., Blazer, V. S., Walsh, H. L., & Wagner, T. (2025). Factors Influencing the Prevalence of Hyperpigmented Melanistic Lesions in Smallmouth Bass Micropterus dolomieu in the Susquehanna River Basin, Pennsylvania. *Journal of Fish Diseases,**48*, e14033. 10.1111/jfd.14033 PMC11646966 39440689

[CR81] Şen, B., Che-Castaldo, C., Krumhardt, K. M., Landrum, L., Holland, M. M., LaRue, M. A., Long, M. C., Jenouvrier, S., & Lynch, H. J. (2023). Spatio-temporal transferability of environmentally-dependent population models: Insights from the intrinsic predictabilities of Adélie penguin abundance time series. *Ecological Indicators,**150*, 110239.

[CR82] Shah, N. W., Baillie, B. R., Bishop, K., Ferraz, S., Högbom, L., & Nettles, J. (2022). The effects of forest management on water quality. *Forest Ecology and Management,**522*, 120397.

[CR83] Simon, T. P., & Burskey, J. L. (2016). Deformity, erosion, lesion, and tumor occurrence, fluctuating asymmetry, and population parameters for bluntnose minnow (Pimephales notatus) as indicators of recovering water quality in a Great Lakes area of concern, USA. *Archives of Environmental Contamination and Toxicology,**70*, 181–191. 26729349 10.1007/s00244-015-0254-4

[CR84] Simon, T. P., & Emery, E. B. (1995). Modification and assessment of an index of biotic integrity to quantify water resource quality in great rivers. *Regulated Rivers: Research & Management,**11*, 283–298.

[CR85] Sket, B., & Trontelj, P. (2008). *Global diversity of leeches (Hirudinea) in freshwater*. Springer.

[CR86] Smalling, K. L., Devereux, O. H., Gordon, S. E., Phillips, P. J., Blazer, V. S., Hladik, M. L., Kolpin, D. W., Meyer, M. T., Sperry, A. J., & Wagner, T. (2021). Environmental and anthropogenic drivers of contaminants in agricultural watersheds with implications for land management. *Science of the Total Environment,**774*, 145687. 33609846 10.1016/j.scitotenv.2021.145687

[CR87] Smalling, K. L., Romanok, K. M., Bradley, P. M., Morriss, M. C., Gray, J. L., Kanagy, L. K., Gordon, S. E., Williams, B. M., Breitmeyer, S. E., Jones, D. K., et al. (2023). Per-and polyfluoroalkyl substances (PFAS) in United States tapwater: Comparison of underserved private-well and public-supply exposures and associated health implications. *Environment International,**178*, 108033. 37356308 10.1016/j.envint.2023.108033

[CR88] Soranno, P. A., Cheruvelil, K. S., Webster, K. E., Bremigan, M. T., Wagner, T., & Stow, C. A. (2010). Using landscape limnology to classify freshwater ecosystems for multi-ecosystem management and conservation. *BioScience,**60*, 440–454.

[CR89] Starliper, C., Blazer, V., Iwanowicz, L., & Walsh, H. L. (2013). Microbial isolates in diseased fishes, primarily smallmouth bass (Micropterus dolomieu), within the Chesapeake Bay drainage in 2009–2011. *Proceedings of the West Virginia Academy of Science,**85*, 18–32.

[CR90] Stranko, S., Boward, D., Kilian, J., Millard, C., Becker, A., Gauza, R., Schenk, A., Roseberry-Lincoln, A., & O’Connor, M. (2007). *Sampling manual: field protocols*. Maryland Department of Natural Resources, Monitoring and Non-Tidal Assessment Division: Technical report.

[CR91] Sures, B., Nachev, M., Selbach, C., & Marcogliese, D. J. (2017). Parasite responses to pollution: what we know and where we go in ‘Environmental Parasitology’. *Parasites & Vectors,**10*, 1–19. 28166838 10.1186/s13071-017-2001-3 PMC5294906

[CR92] Turner, M. G. (2005). Landscape ecology: what is the state of the science? *The Annual Review of Ecology, Evolution, and Systematics,**36*, 319–344.

[CR93] United States Department of Agriculture. (2012). *Assessment of the Effects of Conservation Practices on Cultivated Cropland in the Upper Mississippi River Basin*. United States Department of Agriculture National Resources Conservation Services, Conservation Effects Assessment Project Report. https://www.nrcs.usda.gov/publications/ceap-crop-2010-Upper-MRB-full.pdf.

[CR94] USEPA. (2016). *Aquatic Resources Monitoring- Aquatic Indicators*. US Environmental Protection Agency. https://archive.epa.gov/nheerl/arm/web/html/indicators.html. Accessed 11-Nov-2023.

[CR95] USEPA. (2019a). *National Rivers and Streams Assessment 2018/19 Field Operations Manual, Non-Wadeable*. US Environmental Protection Agency. https://www.epa.gov/national-aquatic-resource-surveys/national-rivers-streams-assessment-2018-19-field-operations-0.

[CR96] USEPA. (2019b).* National Rivers and Streams Assessment 2018/19 Field Operations Manual, Wadeable*. US Environmental Protection Agency. https://www.epa.gov/national-aquatic-resource-surveys/national-rivers-streams-assessment-2018-19-field-operations.

[CR97] Vidal, L. B. (2008). *Fish as ecological indicators in Mediterranean freshwater ecosystems*. Ph.D. thesis, Universitat de Girona. https://www.tdx.cat/bitstream/handle/10803/7873/Tllb1de1.pdf.

[CR98] Vidal-Martinez, V. M., Pech, D., Sures, B., Purucker, S. T., & Poulin, R. (2010). Can parasites really reveal environmental impact? *Trends in Parasitology,**26*, 44–51. 19945346 10.1016/j.pt.2009.11.001

[CR99] Virginia Commonwealth University. (2023). *INSTAR - Healthy Waters*. http://instar.vcu.edu/. Accessed 22-Nov-2023.

[CR100] Virginia Department of Wildlife Resources. (2020). *A Decade of Fish Disease and Mortality Investigations*. https://dwr.virginia.gov/. Accessed 11-Nov-2023.

[CR101] Volta, P., Tremolada, P., Neri, M. C., Giussani, G., & Galassi, S. (2009). Age-dependent bioaccumulation of organochlorine compounds in fish and their selective biotransformation in top predators from Lake Maggiore (Italy). *Water, Air, and Soil Pollution,**197*, 193–209.

[CR102] Wagner, T., McLaughlin, P., Smalling, K., Breitmeyer, S., Gordon, S., & Noe, G. B. (2022). The statistical power to detect regional temporal trends in riverine contaminants in the Chesapeake Bay Watershed, USA. *Science of The Total Environment,**812*, 152435. 34942241 10.1016/j.scitotenv.2021.152435

[CR103] Walsh, H. L., Rafferty, S. D., Gordon, S. E., & Blazer, V. S. (2022). Reproductive health and endocrine disruption in smallmouth bass (Micropterus dolomieu) from the Lake Erie drainage, Pennsylvania, USA. *Environmental Monitoring and Assessment,**194*, 1–19. 10.1007/s10661-021-09654-2 PMC8643298 34862922

[CR104] Webster, P. C. (2020). *Impervious Cover Thresholds of the North Carolina Piedmont Fish Assemblage*. Master’s thesis, The University of North Carolina at Charlotte.

[CR105] Weiss-Lehman, C. P., Werner, C. M., Bowler, C. H., Hallett, L. M., Mayfield, M. M., Godoy, O., Aoyama, L., Barabás, G., Chu, C., Ladouceur, E., et al. (2022). Disentangling key species interactions in diverse and heterogeneous communities: A Bayesian sparse modelling approach. *Ecology Letters,**25*, 1263–1276. 35106910 10.1111/ele.13977 PMC9543015

[CR106] Wieczorek, M., Jackson, S., Schwarz, G. (2018). *Select Attributes for NHDPlus Version 2.1 Reach Catchments and Modified Network Routed Upstream Watersheds for the Conterminous United States (ver. 4.0, August 2023)*. 10.5066/F7765D7V.

[CR107] Willacker, J. J., Eagles-Smith, C. A., & Blazer, V. S. (2020). Mercury bioaccumulation in freshwater fishes of the Chesapeake Bay watershed. *Ecotoxicology,**29*, 459–484. 32239332 10.1007/s10646-020-02193-5

[CR108] Winslow, L. A., Chamberlain, S., Appling, A. P., Read, J. S. (2016). sbtools: A Package Connecting R to Cloud-based Data for Collaborative Online Research. *The R Journal*, *8*. https://journal.r-project.org/archive/2016-1/winslow-chamberlain-appling-etal.pdf.

[CR109] Wu, P., Bishop, K., von Brömssen, C., Eklöf, K., Futter, M., Hultberg, H., Martin, J., & Åkerblom, S. (2018). Does forest harvest increase the mercury concentrations in fish? Evidence from Swedish lakes. *Science of the Total Environment,**622*, 1353–1362. 10.1016/j.scitotenv.2017.12.075 29890601

[CR110] Yeom, D.-H., Chung, K.-H., Kim, Y.-H., & Adams, S. M. (2009). Ecological health and causal assessment of fish communities experiencing multiple stressors in Gap Stream, South Korea. *Toxicology and Environmental Health Sciences,**1*, 97–108.

