# Supplementary material for: Deformity, erosion, lesion, tumor, and parasite (DELT) anomalies in fish communities of the Chesapeake Bay watershed, USA: a regional assessment and potential landscape drivers
Source: Environ Monit Assess. 2025 Aug 8;197(9):998. doi: 10.1007/s10661-025-14412-9 (PMC12334471; doi:10.1007/s10661-025-14412-9)
Supplement: Supplementary file 1 — (pdf 3703 KB) [file 10661_2025_14412_MOESM1_ESM.pdf]

Deformity, erosion, lesion, tumor, and parasite (DELT) anomalies in fish communities of the Chesapeake Bay watershed, USA: A regional assessment and potential landscape drivers

Sara E. Breitmeyer[1], Paul McLaughlin[2], Vicki S. Blazer[3], Gregory B. Noe[4], Kelly L. Smalling[5], Timothy Wertz[6], and Tyler Wagner[7]

[1] *U.S. Geological Survey, Pennsylvania Water Science Center, Downingtown, PA 19335, USA, ORCID: 0000-0003-0609-1559, sbreitmeyer@usgs.gov*
[2] *Pennsylvania Cooperative Fish and Wildlife Research Unit, The Pennsylvania State University, University Park, PA, USA, ORCID: 0000-0001-8344-6793, paulsmclaughlin@gmail.com*
[3] *U.S. Geological Survey, Eastern Ecological Science Center Leetown Research Laboratory, Kearneysville, WV 25430, USA, ORCID: 0000-0001-6647-9614, vblazer@usgs.gov*
[4] *U.S. Geological Survey, Florence Bascom Geoscience Center, Dover, DE 19901, USA, ORCID: 0000-0002-6661-2646, gnoe@usgs.gov*
[5] *U.S. Geological Survey, New Jersey Water Science Center, Lawrenceville, NJ 08648, USA, ORCID: 0000-0002-1214-4920, ksmall@usgs.gov*
[6] *Pennsylvania Department of Environmental Protection, Bureau of Clean Water, Harrisburg, PA 17101, USA, ORCID: 0000-0003-0878-579X, twertz@pa.gov*
[7] *U.S. Geological Survey, Pennsylvania Cooperative Fish and Wildlife Research Unit, The Pennsylvania State University, University Park, PA, USA, ORCID: 0000-0003-1726-016X, txw19@psu.edu*

*__Corresponding author__: *sbreitmeyer@usgs.gov*

Table A1: Summary table of collection agency DELT protocols. EPA NRSA (U.S. Environmental Protection Agency National Rivers and Streams Assessment); INSTAR (Virginia Interactive Stream Assessment); VADEQ (Virginia Department of Environmental Quality); VDWR (Virginia Department of Wildlife Resources); MDDNR (Maryland Department of Natural Resources); Montgomery County; Montgomery County Department of Environmental Protection; PADEP (Pennsylvania Department of Environmental Protection)

Attribute	EPA NRSA, INSTAR, VADEQ, & VDWR	MDDNR & Montgomery County	PADEP
Collection Sites	Both random and targeted; The survey design consists of two separate designs to estimate current status and change in status by resampling past sites and sampling new sites. Sites included represent various Strahler order categories and nine aggregated ecoregions. In addition, a minimum of 20 sites (Resample and New) was guaranteed in each state and a maximum of 75 sites was the limit for an individual state. Approximately 10% of the total sites are scheduled for repeated sampling (revisit sites) in the same year of each two year field cycle.	Both random and targeted; Primary sites are selected randomly for broad-scale assessment. However, some targeted sites used to answer important management questions. *For Montgomery County only: each year 100 sites per region are monitored, monitoring occurs on a 5-year rotation, regions are cycled through annually.	Both random and targeted; Specific site locations will first be determined by the type of study and its design (e.g., cause and effect, aquatic life use assessment) secondly by access feasibility (e.g., public access, private landowner permission, distance to stream etc.) and lastly by representative habitat.

Table A1: Summary table of collection agency DELT protocols *(continued)*

Attribute	EPA NRSA, INSTAR, VADEQ, & VDWR	MDDNR & Montgomery County	PADEP
Fish communities	The fish sampling method is designed to provide a representative sample of the fish community, collecting all but the rarest fish taxa inhabiting the site. This is intended to accurately represent species richness, species guilds, relative abundance, size, and presence of anomalies.	The entire site is thoroughly electrofished, bank to bank, including backwater areas, sloughs, and shallows, making an equal attempt to capture every fish observed. However, fish with a total body length less than 30 mm need not be collected. Continuous rather than intermittent electrofishing is used to avoid bias introduced by selective displacement. The objective is to acquire a representative sample of the overall fish community (i.e., fish assemblage). The collection protocols should be able to consistently characterize most species present.	Fish collection methods are designed to collect a representative sample of the fish assemblage at each sampling station. All fish are identified to the lowest taxonomic level possible, enumerated, and examined for the presence of external anomalies. A variety of electrofishing gear may be used to effectively sample the diversity of habitats within a stream or river reach. The selection of the appropriate gear is based on habitat characteristics, flow conditions, time of year, and sampling objectives.
Seasonal collection	Beginning of June through end of September for most regions; select regions started in April and ended in November.	Electrofishing occurs during the Summer Index Period (June 1-September 30). This time period was chosen to characterize fish communities during the low flow period. Sampling during this period is also advantageous because spawning effects are minimized. Less than ten percent of DELT collection for Montgomery County occurred in October or November.	June 1st- September 30th sampling season
References	All four agencies are based on EPA methods: (Boatable) USEPA (2019) and (Wadeable) USEPA (2019). For VADEQ-specific information see: https://www.deq.virginia.gov/our-programs/water/water-quality/monitoring/probabilistic-monitoring	Stranko et al. (2007); *Montgomery County: https://mygreenmontgomery.org/2020/monitoring-montgomery-countys-streams/	Lookenbill and Whiteash (2021)

Table A1: Summary table of collection agency DELT protocols *(continued)*

Attribute	EPA NRSA, INSTAR, VADEQ, & VDWR	MDDNR & Montgomery County	PADEP

Table A2: Percentage of individual fish observed for DELT, in different seasons, by collection agency. A total of 577,266 individual fish were observed for DELT across all collection agencies and a total of three seasonal sampling scenarios are defined as Spring (February, March, April, May), Summer (June, July, August, September), and Fall (October, November, December, January)

Collection agency	Spring (%)	Summer (%)	Fall (%)
EPA NRSA	0.0	97.2	2.8
INSTAR	16.6	52.8	30.6
MDDNR	0.0	100.0	0.0
Montgomery County	0.0	90.3	9.7
PADEP	0.0	100.0	0.0
VADEQ	0.2	72.9	26.9
VDWR	7.0	17.3	75.7
**All collection agencies**	**1.4**	**84.1**	**14.5**

Table A3: Pennsylvania Department of Environmental Protection (PADEP) deformity (D), erosion (E), lesion (L), tumor (T), or parasite (P) anomaly descriptions (Lookenbill and Whiteash 2021) used to categorize DELT from all collection agencies

Common name	Category	Description
Black grub (Black spot)	P	Small black cyst approximately one millimeter in diameter, caused by a larval trematode
External parasite	P	Ectoparasite attached to body, fins or gills (Leech, Fish louse, Anchor parasite etc.)
Deformities	D	Body structure is abnormal, ex. curvature of the spine
Fungal infection	L	Fungal infections may include a fuzzy (cotton) appearance with discolored areas or lesions
Melanistic area	L	Darkly pigmented spot(s), not raised from the skin
Open sore	L	Lesions, (Note any mucus or necrotic tissue that may be in or around the sore)
Raised red sore	L	A bulging or bubbled red sore
Scales hemorrhagic	L	Evidence of bleeding or hemorrhaging at base of scales
Tumor	T	Unusual mass or fatty growth
White cysts	P	Usually a small crème-colored wart or bubble appearance
Caudal cysts	P	Small cysts specifically found on caudal peduncle, or at the base of anal/dorsal fins
Emaciated	D	Body thin and lacks normal robustness, "starved"
Eye cloudy	L	Eye cloudy or opaque
Eye exophthalmic	L	Eye bulging out of socket
Eye hemorrhagic	L	Eye bleeding or has evidence of burst blood vessels
Eye deteriorated	L	Eye deteriorated, obviously blind, eye missing completely
Fins eroded	E	Erosion of the fin (make note of spawning, as some species will erode fins during this active time)
Fins hemorrhagic	E	Evidence of bleeding or hemorrhaging of fins
Fins frayed	E	Fins appear worn out and stressed (make note of spawning)
Gills eroded	E	Necrotic tissue or eroded to the point that sections of the gill filaments are missing
Gills frayed	E	Gill lamellae appear worn out and stressed
Gill spots	P	Small white spots throughout the lamellae
Gill cysts	P	Cyst attached to gills, usually a small crème-colored wart or bubble appearance

Table A4: Deformity (D), erosion (E), lesion (L), tumor (T), parasite (P), and other (O) anomaly crosswalk between various collection agencies, used to summarize overall percentages of observed DELT

Category	EPA NRSA	INSTAR	MDDNR	Montgomery County	VADEQ	VDWR	PADEP
Deformity	Blackening	Abnormal operculum	Body Shape	Body Shape	Deformed or deformities	Deformed or deformities	Deformed or deformities
	Irregular fin, rays, or scales	Deformed or deformities	Deformities of Mandible	Deformities of Mandible	Emaciated	Emaciated	Emaciated
	Shortened operculum	Eye deformity	Deformities of Vertebral Column	Deformities of Vertebral Column			
	Skeletal deformities	Facial anomaly	Fin Deformed or Missing	Fin Deformed or Missing			
		Lip deformity	Scale Deformities	Scale Deformities			
		Missing barbels	Swelling of the Anus				
		Missing operculum					
		No caudal fin					
		Paralyzed					
		Scoliosis					
		Stubby					
		Stunted					
		Swollen stomach					
Erosion	Eroded fins	Fin rot	Eroded fins	Eroded fins	Eroded fins	Eroded fins	Eroded fins
	Tail wound	Fin Cloudiness	Fin rot	Fins frayed			
					Bird wound	Fins hemorrhagic	
						Gills eroded	
						Gills frayed	
Lesion	Bleeding or reddening	Abrasion	Cataract	Cataract	Exopthalmia (pop eye)	Exopthalmia (pop eye)	Exopthalmia (pop eye)

Table A4: DELTPO anomaly crosswalk between various collection agencies *(continued)*

Category	EPA NRSA	INSTAR	MDDNR	Montgomery County	VADEQ	VDWR	PADEP
	Blisters	Exopthalmia (pop eye)	Cut	Cut	Fungal infection	Eye Missing	Eye Cloudiness
	Excessive mucus	Fungal infection	Depression into the Orbits	Exopthalmia (pop eye)	Lesion	Fungal infection	Eye deteriorated
	Fungal infection	Head wound	Exopthalmia (pop eye)	Eye Cloudiness		Hooking Injury	Eye Hemorrhage
	Lesion	Infection	Eye Cloudiness	Eye Hemorrhage		Lesion	Fungal infection
	Missing organs (eye, fin)	Lesion	Eye Hemorrhage	Eye Missing		Melanistic area	Lesion
	Ulcerous sores	Missing eye	Eye Missing	Fungal infection		Sore	Melanistic area
	Sore	Fungal infection	Hemorrhaging			Raised red sore	
	Ulcer	Hemorrhaging	Hooking Injury			Scales hemor-rhagic	
	Wound	Hooking Injury	Injury				
			Raised Scales	Raised Scales			
			Red Spot	Red Spot			
			Ulcerations/ Lesions	Ulcerations/ Lesions			
Tumor	Tumor	Tumor	Growths/ Cysts	Growths/ Cysts	Tumor	Tumor	Tumor
Parasite	Cyst	Black grub (Black spot)	Anchor Worm	Anchor Worm	Anchor Worm	Anchor Worm	Black grub (Black spot)
	White spots	Cyst	Black grub (Black spot)	Black grub (Black spot)	Black grub (Black spot)	Black grub (Black spot)	Caudal cysts
	Glochidia	Ich	Ich	Leech(es)	Gill parasite	Gill cysts	
	Grub	Leech(es)	Leech(es)	Parasite	Ich	Gill spots	
	Leech(es)	Visible External Parasites	Visible External Parasites	Yellowgrub	Leech(es)	Visible External Parasites	
	Lernia (parasite)	Yellowgrub		Parasite	White cysts		

Table A4: DELTPO anomaly crosswalk between various collection agencies *(continued)*

Category	EPA NRSA	INSTAR	MDDNR	Montgomery County	VADEQ	VDWR	PADEP
	Parasite			Grub			
	Scale parasite						
	Yellowgrub						
Other	Other	Discoloration	Other	Other	Albino		
	Pickled	Other			Eye parasite	Eye parasite	

[1]In the field collection of DELT, anomalies are not always categorized by collection agency into the D,E,L,T, P, or O category. The authors did the best to crosswalk agency-specific DELT categorizations into each of these categories to present a summary of percentages of DELTPO for the overall dataset. Where agencies provided a category for their "O" observations not categorized by PADEP, that category was adopted. The EPA did not record the specific types of DELT in their data, but only the presence or absence of DELT. The DELT for EPA included here were detailed in their field standard operating procedure (USEPA 2019). [EPA NRSA, U.S. Environmental Protection Agency National Rivers and Streams Assessment; INSTAR, Virginia Interactive Stream Assessment; MDDNR, Maryland Department of Natural Resources; VADEQ, Virginia Department of Environmental Quality; VDWR, Virginia Department of Wildlife Resources]

Table A5: Landscape predictor mosaic categories, definitions, and units **(for the total upstream watershed area)**

Landscape Predictor	Mosaic Category	Mosaic Sub-category	Definition	Units	Temporal Range/Year	Data Source
Elevation	Freshwater	Drainage network	Mean land elevation in the total upstream watershed	m	2012	(Wieczorek et al. 2018)
Stream density	Freshwater	Drainage network	Stream density	km stream/sq-km	2012	(Wieczorek et al. 2018)
Stream slope	Freshwater	Drainage network	Average flowline slope in the total upstream watershed	percentage	2012	(Wieczorek et al. 2018)
Basin slope	Freshwater	Drainage network	Total upstream watershed's mean slope	percentage	2012	(Wieczorek et al. 2018)
Artificial flowlines	Freshwater	Drainage network	Percentage of all flowline reach lengths that are artificial paths (type of National Hydrography Dataset (NHD) flowline) in the total upstream watershed	percentage	2012	(Wieczorek et al. 2018)
Canal flowlines	Freshwater	Drainage network	Percentage of all flowline reach lengths that are a canal or ditch (type of NHD flowline) in the total upstream watershed	percentage	2012	(Wieczorek et al. 2018)
Connector flowlines	Freshwater	Drainage network	Percentage of all flowline reach lengths that are a connector (type of NHD flowline) in the total upstream watershed	percentage	2012	(Wieczorek et al. 2018)
Pipeline flowlines	Freshwater	Drainage network	Percentage of all flowline reach lengths that are a pipeline (type of NHD flowline) in the total upstream watershed	percentage	2012	(Wieczorek et al. 2018)

Table A5: Landscape predictor mosaic categories, definitions, and units (for the total upstream watershed area) *(continued)*

Landscape Predictor	Mosaic Category	Mosaic Sub-category	Definition	Units	Temporal Range/Year	Data Source
Base flow index	Freshwater	Groundwater	Base Flow Index, ratio of base flow to total streamflow as a percentage. The sustained, slowly varying component of streamflow, attributed to ground-water discharge in the total upstream watershed	percentage	2003	(Wieczorek et al. 2018)
Water table depth	Freshwater	Groundwater	Average depth from land surface to the seasonally high water table in the total upstream watershed	feet	n/a	(Wieczorek et al. 2018)
Subsurface flow contact time	Freshwater	Groundwater	Subsurface flow contact time index, estimates the number of days that infiltrated water resides in the saturated subsurface zone of the basin before discharging into the stream in the total upstream watershed	days	2014	(Wieczorek et al. 2018)

Table A5: Landscape predictor mosaic categories, definitions, and units (for the total upstream watershed area) *(continued)*

Landscape Predictor	Mosaic Category	Mosaic Sub-category	Definition	Units	Temporal Range/Year	Data Source
Overland flow	Freshwater	Groundwater	Dunne overland flow in the total upstream watershed. Dunne overland flow is a mechanism for runoff generation that occurs when soils cannot absorb water, such as when rain or meltwater flows over saturated soils. This results in saturation excess overland flow, which occurs due to surface water input on areas already saturated. Infiltrating water completely saturates the soil profile resulting in the water table rising to the surface. Generally higher in humid regions	percentage	n/a	(Wieczorek et al. 2018)
Stream order	Freshwater	Runoff	Stream Strahler order	numeric order (categorical)	2012	(Wieczorek et al. 2018)
Precipitation	Freshwater	Precipitation	Mean annual average precipitation in the total upstream watershed, from 800-meter Parameter-Elevation Regressions on Independent Slopes Model (PRISM)	mm/yr	1989-2015	(Wieczorek et al. 2018)
Runoff	Freshwater	Runoff	Mean runoff from the Water Balance Model, in the total upstream watershed. Generated by all types of mechanisms (surface, subsurface, and baseflow)	mm/yr	2000-2014	(Wieczorek et al. 2018)
Dam density	Human	Dams, road networks	Dam density, built on or before 2013, in the total upstream watershed	dams/sq-km	2013	(Wieczorek et al. 2018)

Table A5: Landscape predictor mosaic categories, definitions, and units (for the total upstream watershed area) *(continued)*

Landscape Predictor	Mosaic Category	Mosaic Sub-category	Definition	Units	Temporal Range/Year	Data Source
Road density	Human	Dams, road networks	Road density (all road types). Defined as the length of road divided by the total upstream watershed area	km roads/sq-km	2012	(Wieczorek et al. 2018)
Road/stream crossing	Human	Dams, road networks	Road & stream intersections density in the total upstream watershed	crossings/sq-km	2012	(Wieczorek et al. 2018)
Cropland	Human	Land use	Cropland in the total upstream watershed (barren, herbaceous, and orchard/vineyard-barren, -herbaceous, and -scrub/shrub)	percentage	2013-2018	(Chesapeake Bay Program 2023)
Extractive land	Human	Land use	Extractive land (barren and impervious lands due to surface mining operations) in the total upstream watershed	percentage	2013-2018	(Chesapeake Bay Program 2023)
Harvested forest	Human	Land use	Harvested Forest (barren and herbaceous) in the total upstream watershed	percentage	2013-2018	(Chesapeake Bay Program 2023)
Pasture	Human	Land use	Pasture/Hay (barren, herbaceous, and scrub/shrub lands) in the total upstream watershed	percentage	2013-2018	(Chesapeake Bay Program 2023)
Impervious land	Human	Land use	Impervious land (land with roads and structures, other impervious land, and solar fields) in the total upstream watershed	percentage	2013-2018	(Chesapeake Bay Program 2023)
Phyto cover crop	Human	Land use	High-phytoestrogen plant crop cover in the total upstream watershed, mean of 2002 & 2008 -2016.	Percentage	2002-2016	(Gordon et al. 2017)

Table A5: Landscape predictor mosaic categories, definitions, and units (for the total upstream watershed area) *(continued)*

Landscape Predictor	Mosaic Category	Mosaic Sub-category	Definition	Units	Temporal Range/Year	Data Source
CAFO animal density	Human	Land use	Confined Animal Feeding Operation (CAFO) animal density in the total upstream watershed	CAFO animals/sq-km	2016	(Gordon et al. 2017)
Landfill density	Human	Land use	Landfill density in the total upstream watershed	landfills/sq-km	2015	(Gordon et al. 2017)
TRI facility density	Human	Land use	Toxic Release Inventory (TRI) facility density in the total upstream watershed	facility/sq-km	2015	(Gordon et al. 2017)
Biosolid N	Human	Land use	Mean nitrogen from biosolids in the total upstream watershed	kg/sq-km	2003-2013	(Gordon et al. 2017)
Manure N	Human	Land use	Total nitrogen from manure in the total upstream watershed, mean of 2002, 2007, & 2012	kg/sq-km	2002-2012	(Gordon et al. 2017)
CSO density	Human	Land use	Combined Sewer Overflow (CSO) density in the total upstream watershed	CSO/sq-km	2016	(Gordon et al. 2017)
Public WWTP density	Human	Land use	Publicly Owned Treatment Works (POTW) Discharge Monitoring Report (DMR) outfall monitoring location density in the total upstream watershed	facility/sq-km	2016	(Gordon et al. 2017)
Local septic density	Human	Land use	Septic facility density, including individual housing in the total upstream watershed	facility/sq-km	2010-2014	(Gordon et al. 2017)
Large septic density	Human	Land use	EPA large scale septic system (might be used for non-sanitary purposes, primarily at industrial & automotive areas) density in the total upstream watershed	facility/sq-km	2016	(Gordon et al. 2017)

Table A5: Landscape predictor mosaic categories, definitions, and units (for the total upstream watershed area) *(continued)*

Landscape Predictor	Mosaic Category	Mosaic Sub-category	Definition	Units	Temporal Range/Year	Data Source
Irrigated ag	Human	Land use	Irrigated agriculture in the total upstream watershed, mean of 2002, 2007, & 2012. Represents the percent of the entire watershed area that is classified as irrigated agriculture. From the U.S. Geological Survey Moderate Resolution Imaging Spectroradiometer (MODIS) 250-m data	percentage	2002-2012	(Wieczorek et al. 2018)
Pesticide use	Human	Land use	Combined fungicide, herbicide, insecticide, & nematicide use on agricultural land in the total upstream watershed	kg/sq-km	2009	(Wieczorek et al. 2018)
Housing density	Human	Land use	Historic housing densities in the total upstream watershed	percentage	2010	(Wieczorek et al. 2018)
NPDES facility density	Human	Land use	Major National Pollution Discharge Elimination System (NPDES) facility density in the total upstream watershed	facility/sq-km	2010	(Wieczorek et al. 2018)
FRS facility density	Human	Land use	Facility Registry Service (FRS) facility density in the total upstream watershed	facility/sq-km	2016	(Gordon et al. 2017)
DMR facility density	Human	Land use	Non-Publicly Owned Treatment Works (non-POTW) Discharge Monitoring Report (DMR) outfall monitoring facility density in the total upstream watershed	facility/sq-km	2016	(Gordon et al. 2017)
Hg deposition	Human	National policy, atmospheric deposition	Mean mercury deposited from the atmosphere in the total upstream watershed	ug/sq-km	2003-2014	(Gordon et al. 2017)

Table A5: Landscape predictor mosaic categories, definitions, and units (for the total upstream watershed area) *(continued)*

Landscape Predictor	Mosaic Category	Mosaic Sub-category	Definition	Units	Temporal Range/Year	Data Source
No till	Human	National policy, atmospheric deposition	Land on which no-till practices were used in the total upstream watershed	percentage	2012	(Wieczorek et al. 2018)
Conservation easement	Human	National policy, atmospheric deposition	Land under a conservation easement in the total upstream watershed	percentage	2012	(Wieczorek et al. 2018)

Table A5: Landscape predictor mosaic categories, definitions, and units (for the total upstream watershed area) *(continued)*

Landscape Predictor	Mosaic Category	Mosaic Sub-category	Definition	Units	Temporal Range/Year	Data Source
Ag sed red BMP	Human	National policy, atmospheric deposition	Estimated sediment loss from agricultural fields due to the implementation of a suite of Best Management Practices (BMPs) in the total upstream watershed. Estimated from the National Resource Conservation Service's (NRCS) BMP Scenarios that included structural practices for controlling water/wind erosion and reduced tillage using either no tillage/mulch tillage. Structural practices included: (1) NRCS overland flow control practices (terraces, contour farming, strip-cropping, in-field vegetative barriers, and field borders); (2) NRCS concentrated flow control practices (grassed waterways, grade stabilization structures, diversions, and water and sediment control basins); (3) NRCS and Conservation Reserve Program's edge-of-field buffering and filtering practices (grasses, shrubs, and/or trees strips and riparian herbaceous and riparian forest buffers); and (4) wind erosion control practices (windbreaks/shelterbelts, cross wind trap strips, herbaceous windbreak, and hedgerow planting).	tons/sq-km	2012	(Wieczorek et al. 2018, United States Department of Agriculture 2012)

Table A5: Landscape predictor mosaic categories, definitions, and units (for the total upstream watershed area) *(continued)*

Landscape Predictor	Mosaic Category	Mosaic Sub-category	Definition	Units	Temporal Range/Year	Data Source
Ag TN red BMP	Human	National policy, atmospheric deposition	Estimated average loss of the sum of nitrogen (N) in runoff, water & wind erosion, tile drainage, percolate, subsurface & quick return flow in the total upstream watershed, from the NRCS BMP Scenarios that included structural practices for controlling water/wind erosion, reduced tillage using either no tillage/mulch tillage, and nitrogen management through appropriate timing (e.g., within 3 weeks before planting or 60 days after planting), method (e.g., incorporated or banding/foliar/spot treatment), and appropriate rates of total nitrogen application (including manure). Structural practices included: (1) NRCS overland flow control practices (terraces, contour farming, strip-cropping, in-field vegetative barriers, and field borders); (2) NRCS concentrated flow control practices (grassed waterways, grade stabilization structures, diversions, and water and sediment control basins); (3) NRCS and Conservation Reserve Program's edge-of-field buffering and filtering practices (grasses, shrubs, and/or trees strips and riparian herbaceous and riparian forest buffers); and (4) wind erosion control practices (windbreaks/shelterbelts, cross wind trap strips, herbaceous windbreak, and hedgerow planting).	kg/sq-km	2012	(Wieczorek et al. 2018, United States Department of Agriculture 2012)

Table A5: Landscape predictor mosaic categories, definitions, and units (for the total upstream watershed area) *(continued)*

Landscape Predictor	Mosaic Category	Mosaic Sub-category	Definition	Units	Temporal Range/Year	Data Source
Cons till	Human	National policy, atmospheric deposition	Land on which conservation tillage practices were used in the total upstream watershed	percentage	2012	(Wieczorek et al. 2018)
Conv tillage	Human	National policy, atmospheric deposition	Land on which conventional tillage practices were used in the total upstream watershed	percentage	2012	(Wieczorek et al. 2018)
Cover crop	Human	National policy, atmospheric deposition	Land which is planted to a cover crop (excluding Conservation Reserve Program land) in the total upstream watershed	percentage	2012	(Wieczorek et al. 2018)
N deposition	Human	National policy, atmospheric deposition	Mean total deposition (wet and dry) of nitrogen (oxidized + reduced) in the total upstream watershed	kg-N/sq-km	2002-2012	(Wieczorek et al. 2018)
Forestry activity	Terrestrial	Catchment morphology	Annual information on forestry activity (timber clear-cutting and harvest activities) in the total upstream watershed	percentage	1999-2012	(Wieczorek et al. 2018)
Rock CaO	Terrestrial	Geology	Rock lithological calcium oxide concentration in the total upstream watershed, based on Olson geology types in surface or near surface geology	percentage	2009	(Wieczorek et al. 2018)
Sinkhole density	Terrestrial	Geology	Sinkhole density in the total upstream watershed	depressions/sq-km	2020	(Jones et al. 2021)

Table A5: Landscape predictor mosaic categories, definitions, and units (for the total upstream watershed area) *(continued)*

Landscape Predictor	Mosaic Category	Mosaic Sub-category	Definition	Units	Temporal Range/Year	Data Source
Temperature	Terrestrial	Riparian characteristic	Mean annual average air temperature from 800-meter Parameter-Elevation Regressions on Independent Slopes Model (PRISM), in the total upstream watershed	Celsius	1989-2015	(Wieczorek et al. 2018)
Silt	Terrestrial	Soils	Average silt in soil in the total upstream watershed	percentage	n/a	(Wieczorek et al. 2018)
Soil organic matter	Terrestrial	Soils	Average organic matter content in soil in the total upstream watershed	percentage by weight	n/a	(Wieczorek et al. 2018)
Soil pH	Terrestrial	Soils	Average soil pH in the total upstream watershed	standard unit	n/a	(Wieczorek et al. 2018)
Clay	Terrestrial	Soils	Average clay content in soil in the total upstream watershed	percentage	n/a	(Wieczorek et al. 2018)

Table A6: Landscape predictor variable summary statistics.

Predictor	Mean	Median	Interquartile range	Range, minimum - maximum	Units
Ag Sed Red BMP	5976	462	207	0 - 4357312	tons/sq-km
Artificial Flowlines	1	0	0.7	0 - 100	percentage
Basin Slope	11	7	8	0.1 - 48	percentage
Base Flow Index	48	49	8	26 - 65	percentage
Biosolid N	20	0.4	1	0 - 16330	kg/sq-km
CAFO Animal Density	2038	0	0	0 - 1771444	CAFO animals/sq-km
Canal Flowlines	0.07	0	0	0 - 25	percentage
Clay	22	20	4	12 - 57	percentage
Connector Flowlines	0.03	0	0	0 - 4	percentage
Conservation Easement	1	1	2	0 - 19	percentage
Cons Till	0.8	0.5	1	0 - 13	percentage
Subsurface Flow Contact Time	215	140	166	4 - 13819	days
Conv Till	0.6	0.3	0.6	0 - 13	percentage
Cover Crop	2	1	3	0 - 23	percentage
Cropland	4	0.5	5	0 - 62	percentage
CSO Density	0.002	0	0	0 - 7	CSO/sq-km
Dam Density	0.2	0	0.01	0 - 135	dams/sq-km
DMR Facility Density	0.1	0	0.004	0 - 124	facility/sq-km
Elevation	271	171	277	9 - 1152	m
Extractive Land	0.07	0	0	0 - 6	percentage
Forestry Activity	0.08	0	0.02	0 - 5	percentage
FRS Facility Density	5	0.4	1	0 - 3226	facility/sq-km
Harvested Forest	0.4	0	0.06	0 - 30	percentage
Hg Deposition	88791	8488	469	3716 - 70808017	ug/sq-km
Housing Density	162	24	195	0 - 1743	percentage

Table A6: Landscape predictor variable summary statistics. *(continued)*

Predictor	Mean	Median	Interquartile range	Range, minimum - maximum	Units
Impervious Land	10	6	14	0 - 54	percentage
Irrigated Ag	0.2	0	0.1	0 - 24	percentage
Landfill Density	0.02	0	0	0 - 13	landfills/sq-km
Large Septic Density	0.1	0	0	0 - 107	facility/sq-km
Local Septic Density	137	9	18	0 - 112573	facility/sq-km
Manure N	11878	343	600	0 - 11123441	kg/sq-km
No Till	6	3	9	0 - 47	percentage
NPDES Facility Density	12	0	0	0 - 1898	facility/sq-km
Soil Organic Matter	0.6	0.5	0.1	0.1 - 4	percentage by weight
Overland Flow	3	3	2	1 - 8	percentage
Soil pH	5	5	0.1	5 - 6	standard unit
Pasture	13	10	16	0 - 64	percentage
Pesticide Use	29	15	31	0 - 298	kg/sq-km
Phyto Cover Crop	3	0.7	3	0 - 36	Percentage
Pipeline Flowlines	0.004	0	0	0 - 6	percentage
Precipitation	1119	1125	50	927 - 1413	mm/yr
Public WWTP Density	0.08	0	0	0 - 73	facility/sq-km
Road/Stream Crossing	8	0.8	0.6	0 - 6945	crossings/sq-km
Road Density	4	3	4	0 - 15	km roads/sq-km
Rock CaO	5	3	4	0.4 - 37	percentage
Runoff	414	402	60	250 - 731	mm/yr
Silt	47	49	11	17 - 63	percentage
Sinkhole Density	0.008	0	0	0 - 0.5	depressions/sq-km
Stream Density	0.8	0.8	0.2	0.2 - 2	km stream/sq-km
Stream Order	2	2	2	1 - 7	numeric order (categorical)

Table A6: Landscape predictor variable summary statistics. *(continued)*

Predictor	Mean	Median	Interquartile range	Range, minimum - maximum	Units
Stream Slope	0.01	0.007	0.008	0 - 0.1	percentage
Temperature	12	13	1	7 - 16	Celsius
Ag TN Red BMP	59653	6015	1110	0 - 46541124	kg/sq-km
N Deposition	3161	3243	1197	1962 - 4615	kg-N/sq-km
TRI Facility Density	0.04	0	0	0 - 34	facility/sq-km
Water Table Depth	5	5	0.3	0.9 - 6	feet

Table A7: Observed (naive) DELT occurrence, total fish collected, and total streams sampled for each species in the Chesapeake Bay watershed, 2008 - 2019

Common name (scientific name)	DELT occurrence (proportion observed)	Fish (n)	Streams (n)
Channel Catfish (*Ictalurus punctatus*)	0.1892	888	83
Golden Shiner (*Notemigonus crysoleucas*)	0.0894	705	119
Rock Bass (*Ambloplites rupestris*)	0.0760	7518	273
Smallmouth Bass (*Micropterus dolomieu*)	0.0731	10557	318
Brown Bullhead (*Ameiurus nebulosus*)	0.0558	556	92
Bowfin (*Amia calva*)	0.0541	37	11
Yellow Bullhead (*Ameiurus natalis*)	0.0508	2993	307
River Chub (*Nocomis micropogon*)	0.0491	2790	156
Margined Madtom (*Noturus insignis*)	0.0423	4946	417
Common Shiner (*Luxilus cornutus*)	0.0406	9472	380
Fallfish (*Semotilus corporalis*)	0.0395	9218	429
Common Carp (*Cyprinus carpio*)	0.0375	560	65
Bluespotted Sunfish (*Enneacanthus gloriosus*)	0.0364	467	68
Redfin Pickerel (*Esox americanus*)	0.0337	89	24
Mountain Redbelly Dace (*Chrosomus oreas*)	0.0309	9776	213
Green Sunfish (*Lepomis cyanellus*)	0.0271	11973	442
Creek Chub (*Semotilus atromaculatus*)	0.0269	27794	591
Gizzard Shad (*Dorosoma cepedianum*)	0.0255	431	36
Warmouth (*Lepomis gulosus*)	0.0245	163	61
Pumpkinseed (*Lepomis gibbosus*)	0.0241	2156	277
White Sucker (*Catostomus commersonii*)	0.0237	19294	566
Chain Pickerel (*Esox niger*)	0.0202	495	119
Bluehead Chub (*Nocomis leptocephalus*)	0.0169	11742	464
Northern Hogsucker (*Hypentelium nigricans*)	0.0168	5226	305
Black Crappie (*Pomoxis nigromaculatus*)	0.0160	125	38
Flier (*Centrarchus macropterus*)	0.0159	63	21
Central Stoneroller (*Campostoma anomalum*)	0.0159	17039	322
Torrent Sucker (*Thoburnia rhothoeca*)	0.0153	8815	290
Redbreast Sunfish (*Lepomis auritus*)	0.0147	15200	564
Eastern Creek Chubsucker (*Erimyzon oblongus*)	0.0143	1752	237
Brown Trout (*Salmo trutta*)	0.0136	1619	69
Largemouth Bass (*Micropterus salmoides*)	0.0131	2594	377
Johnny Darter (*Etheostoma nigrum*)	0.0112	2139	255
Bluegill (*Lepomis macrochirus*)	0.0098	14455	631

Table A7: Observed (naive) DELT occurrence, total fish collected, and total streams sampled for each species in the Chesapeake Bay watershed, 2008 - 2019 *(continued)*

Common name (scientific name)	DELT occurrence (proportion observed)	Fish (n)	Streams (n)
Tessellated Darter (*Etheostoma olmstedi*)	0.0092	11945	359
Eastern Blacknose Dace (*Rhinichthys atratulus*)	0.0088	163552	627
Fantail Darter (*Etheostoma flabellare*)	0.0074	22376	474
Longnose Dace (*Rhinichthys cataractae*)	0.0072	21087	401
Potomac Sculpin (*Cottus girardi*)	0.0050	8786	130
Greenside Darter (*Etheostoma blennioides*)	0.0050	2787	106
Yellow Perch (*Perca flavescens*)	0.0049	815	55
Eastern Mudminnow (*Umbra pygmaea*)	0.0043	1165	94
Spottail Shiner (*Notropis hudsonius*)	0.0042	5449	148
Least Brook Lamprey (*Lampetra aepyptera*)	0.0040	250	43
Bluntnose Minnow (*Pimephales notatus*)	0.0039	20098	242
Spotfin Shiner (*Cyprinella spiloptera*)	0.0032	6328	122
Cutlip Minnow (*Exoglossum maxillingua*)	0.0020	5601	171
Pirate Perch (*Aphredoderus sayanus*)	0.0012	1629	214
Satinfin Shiner (*Cyprinella analostana*)	0.0011	3658	216
Eastern Mosquitofish (*Gambusia holbrooki*)	0.0011	1796	132
Rosyside Dace (*Clinostomus funduloides*)	0.0009	29952	459
Blue Ridge Sculpin (*Cottus caeruleomentum*)	0.0008	51195	230
American Eel (*Anguilla rostrata*)	0.0007	8882	423
Swallowtail Shiner (*Notropis procne*)	0.0004	5540	195
White Perch (*Morone americana*)	0.0000	83	8
Redear Sunfish (*Lepomis microlophus*)	0.0000	183	45
Eastern Silvery Minnow (*Hybognathus regius*)	0.0000	462	39

References

Becker, R. A., A. R. Wilks, R. Brownrigg, T. P. Minka, and A. Deckmyn, 2023. maps: Draw Geographical Maps. URL https://CRAN.R-project.org/package=maps.

Chesapeake Bay Program, 2023. Chesapeake Bay Land Use and Land Cover (LULC) Database 2022 Edition. URL https://doi.org/10.5066/P981GV1L.

Gearty, W., and L. A. Jones. 2023. rphylopic: An R package for fetching, transforming, and visualising PhyloPic silhouettes. Methods in Ecology and Evolution **14**:2700–2708.

Gordon, S., D. Jones, B. Williams, and C. Wright, 2017. Potential contaminant sources and other landscape variables summarized for NHDPlus Version 2.1 catchments within the Chesapeake Bay Watershed (ver. 2.0, June 2021). URL https://doi.org/10.5066/F7SQ8ZB3.

Jones, J., D. Doctor, N. Wood, J. Falgout, and N. Rapstine, 2021. Closed depression density in karst regions of the conterminous United States: features and grid data. URL https://doi.org/10.5066/P9EV2I12.

Lookenbill, M., and R. Whiteash, 2021. Water Quality Monitoring Protocols for Streams and Rivers. Technical report, Pennsylvania Department of Environmental Protection, Harrisburg, Pennsylvania. URL https://files.dep.state.pa.us/water/Drinking%20Water%20and%20Facility%20Regulation/WaterQualityPortalFiles/Technical%20Documentation/MONITORING_BOOK.pdf.

R Core Team, 2024. R: A Language and Environment for Statistical Computing. R Foundation for Statistical Computing, Vienna, Austria. URL https://www.R-project.org/.

Stranko, S., D. Boward, J. Kilian, C. Millard, A. Becker, R. Gauza, A. Schenk, A. Roseberry-Lincoln, , and M. O'Connor, 2007. Sampling manual: field protocols. Technical report, Maryland Department of Natural Resources, Monitoring and Non-Tidal Assessment Division.

United States Department of Agriculture. 2012. Assessment of the Effects of Conservation Practices on Cultivated Cropland in the Upper Mississippi River Basin. United States Department of Agriculture National Resources Conservation Services, Conservation Effects Assessment Project Report URL https://www.nrcs.usda.gov/publications/ceap-crop-2010-Upper-MRB-full.pdf.

U.S. Environmental Protection Agency, 2010. Level III and IV Ecoregions of the Continental United States. U.S. EPA Office of Research & Development (ORD) - National Health and Environmental Effects Research Laboratory (NHEERL). https://www.epa.gov/eco-research/level-iii-and-iv-ecoregions-continental-united-states.

USEPA, 2019. National Rivers and Streams Assessment 2018/19 Field Operations Manual, Wadeable. US Environmental Protection Agency. URL https://www.epa.gov/national-aquatic-resource-surveys/national-rivers-streams-assessment-2018-19-field-operations.

Wickham, H. 2016. ggplot2: Elegant Graphics for Data Analysis. Springer-Verlag New York. URL https://ggplot2.tidyverse.org.

Wieczorek, M., S. Jackson, and G. Schwarz, 2018. Select Attributes for NHDPlus Version 2.1 Reach Catchments and Modified Network Routed Upstream Watersheds for the Conterminous United States (ver. 4.0, August 2023). URL https://doi.org/10.5066/F7765D7V.

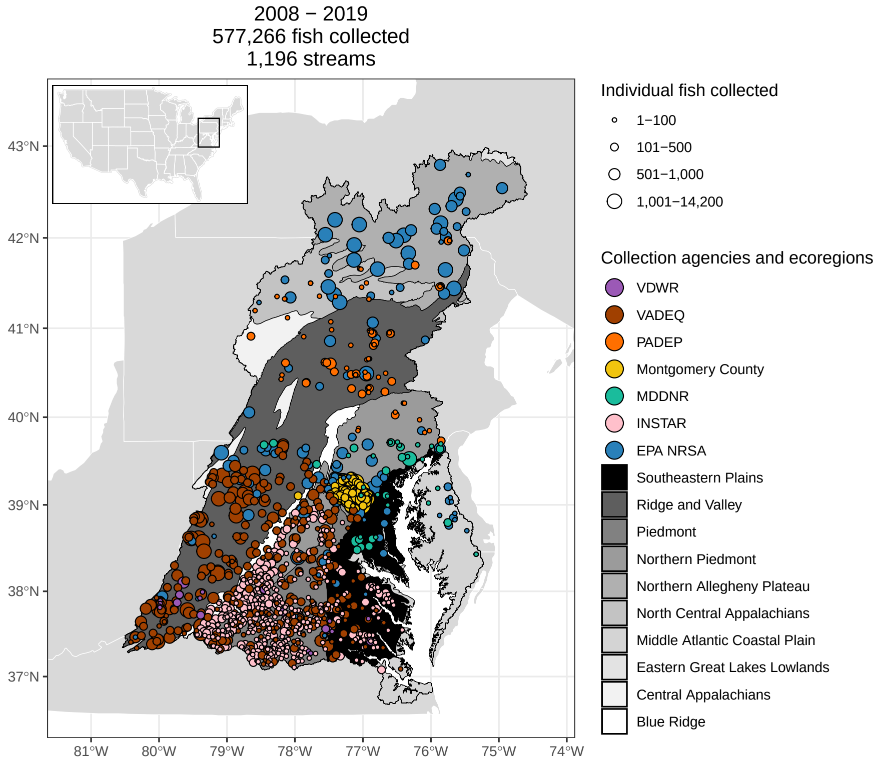

Figure A1: Fish collection streams (n: 1,196), represented by circles, number of individual fish collected by circle size, and collection agencies by colors within the shaded level 3 ecoregions (U.S. Environmental Protection Agency 2010) of the Chesapeake Bay watershed, US. Collection agencies included the U.S. Environmental Protection Agency National Rivers and Streams Assessment (EPA NRSA), Virginia Interactive Stream Assessment (INSTAR), Maryland Department of Natural Resources (MDDNR), Montgomery County Department of Environmental Protection, Pennsylvania Department of Environmental Protection (PADEP), Virginia Department of Environmental Quality (VADEQ), and Virginia Department of Wildlife Resources (VDWR). Fish were collected from nine level 3 ecoregions including the Central and North Central Appalachians, Middle Atlantic Coastal Plain, Northern Allegheny Plateau, Northern Piedmont, Piedmont, Ridge and Valley, Southeastern Plains, and Blue Ridge. The plot utilizes North American Datum of 1983 and a Transverse Mercator projection. State outlines and US map were obtained from the R system R maps package (R Core Team 2024, Becker et al. 2023). The Chesapeake Bay watershed shape outline was obtained from the Chesapeake Bay Program's website (private member 2023).

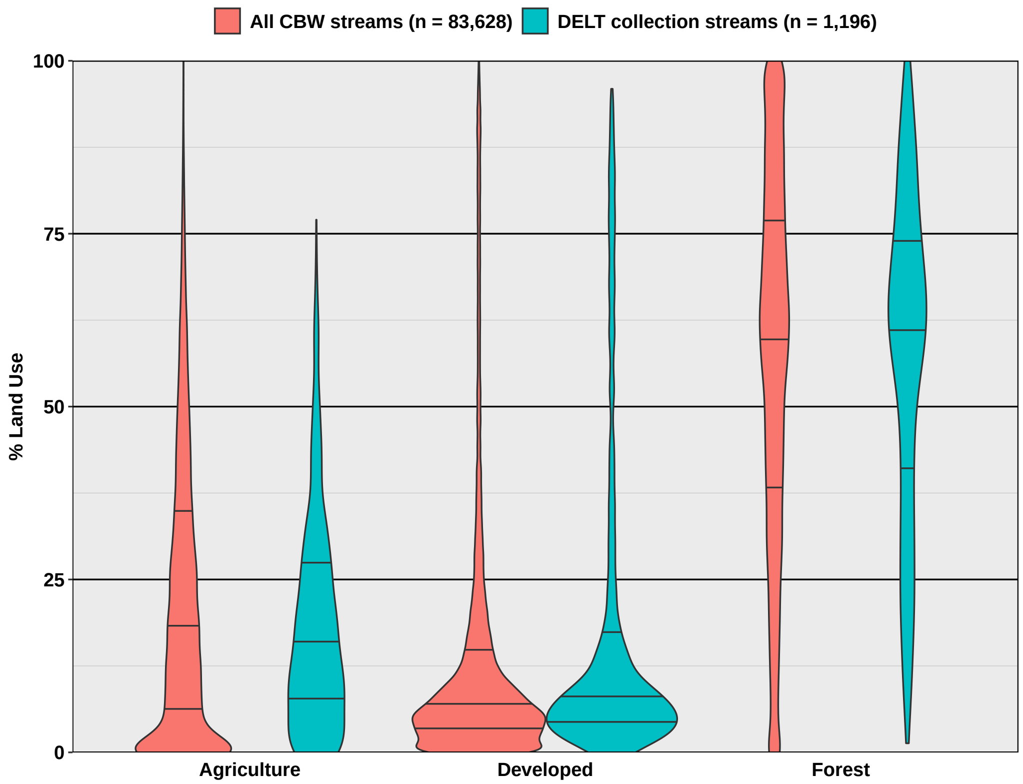

Figure A2: Violin plot of percentages of major land use in all Chesapeake Bay watershed (CBW) streams compared to those in Chesapeake Bay watershed streams where 577,266 individual fish were collected for DELT observations.

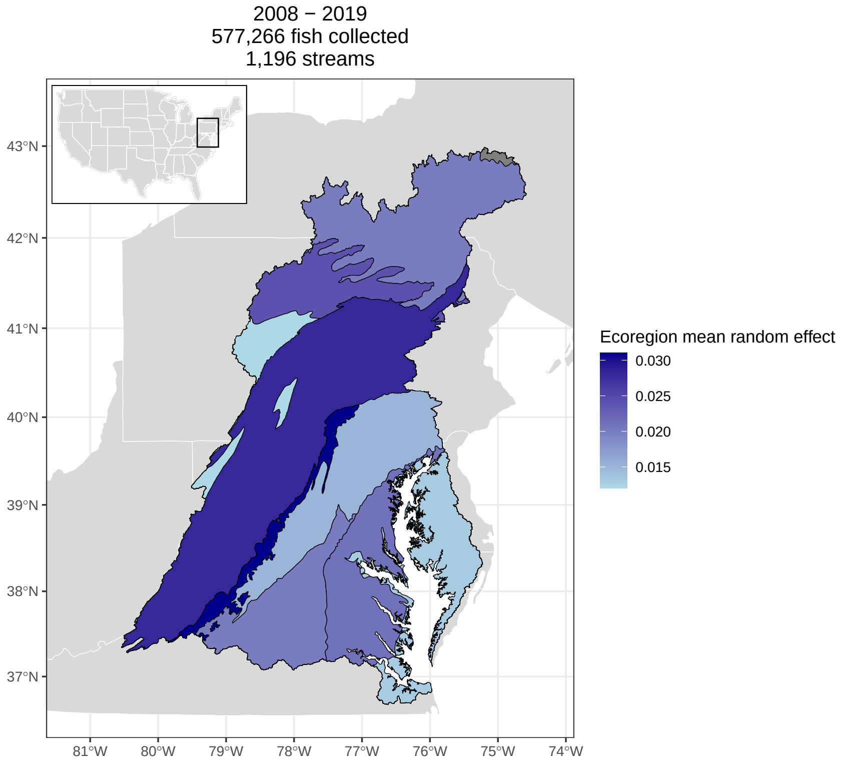

Figure A3: Level 3 ecoregions (U.S. Environmental Protection Agency 2010), which are shaded to represent the ecoregion-specific mean probability obtained from the DELT occurrence model across all fish species in the Chesapeake Bay watershed, US. Fish were collected from nine level 3 ecoregions. The ecoregion-specific mean probability of DELT occurrence (modeled using equation (1)), in ascending order, was as follows: Central Appalachians (0.012, 90% CI = 0.002, 0.032), Middle Atlantic Coastal Plain (0.013, 90% CI = 0.002, 0.034), Northern Piedmont (0.015, 90% CI = 0.003, 0.040), Piedmont (0.020, 90% CI = 0.004, 0.051), Northern Allegheny Plateau (0.020, 90% CI = 0.004, 0.053), Southeastern Plains (0.021, 90% CI = 0.004, 0.055), North Central Appalachians (0.024, 90% CI = 0.005, 0.061), Ridge and Valley (0.028, 90% CI = 0.006, 0.072), and Blue Ridge (0.031, 90% CI = 0.007, 0.082). Refer to Figure A1 for plotted level 3 ecoregion names, fish collection streams, and collection agencies. The plot utilizes North American Datum of 1983 and a Transverse Mercator projection. State outlines and US map were obtained from the R system R maps package (R Core Team 2024, Becker et al. 2023). The Chesapeake Bay watershed shape outline was obtained from the Chesapeake Bay Program's website (private member 2023).

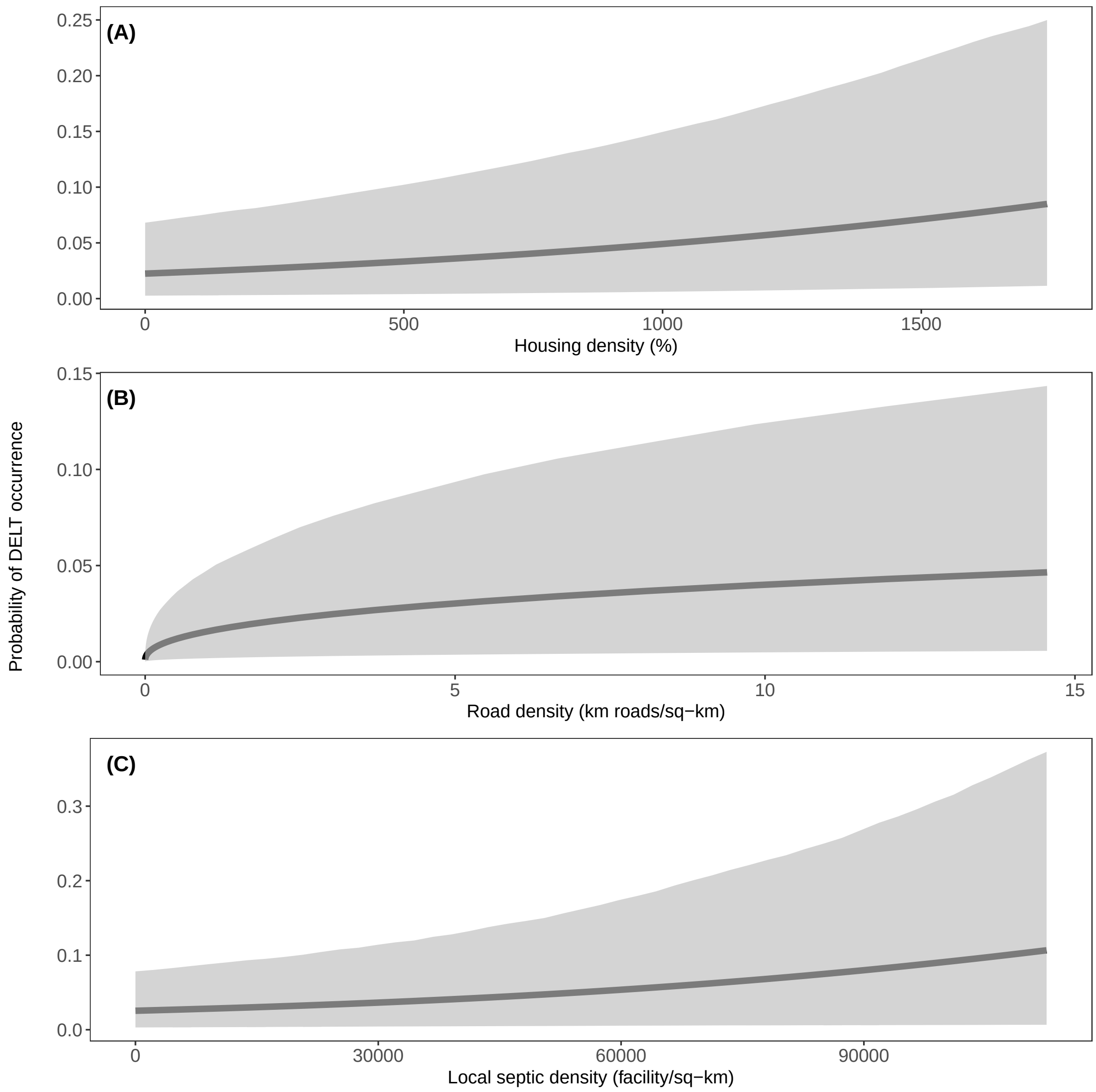

Figure A4: Predicted probability of DELT occurrence across all fish species sampled in the Chesapeake Bay watershed, USA (modeled using equation (2)) as a function of housing density (A), road density (B), and local septic density (C). Solid line is posterior mean and shaded area is 90% credible region. All other predictor variables are held at average values found in this study.

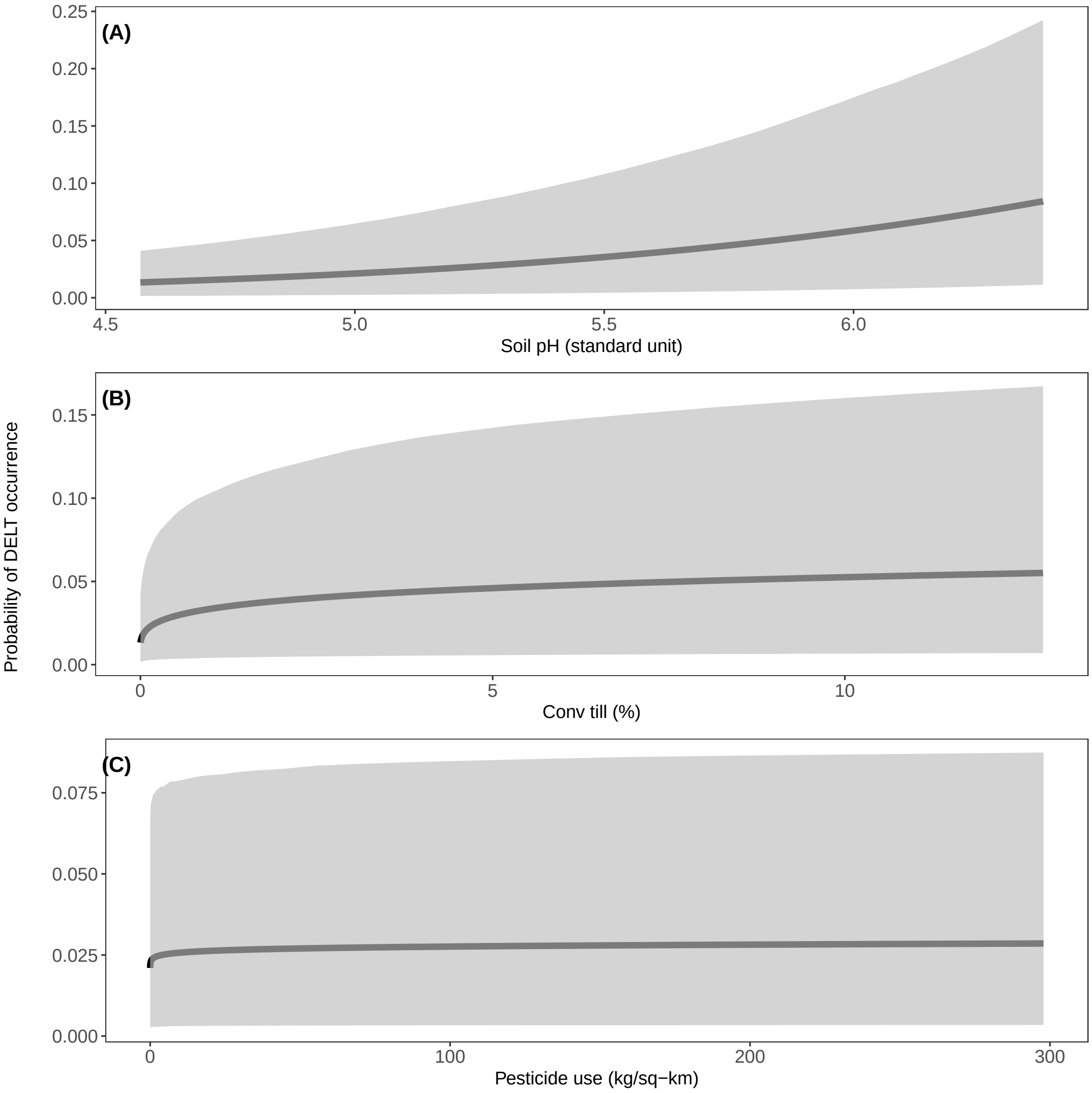

Figure A5: Predicted probability of DELT occurrence across all fish species sampled in the Chesapeake Bay watershed, USA (modeled using equation (2)) as a function of soil pH (A), conventional tillage (B), and estimated pesticide use (C). Solid line is posterior mean and shaded area is 90% credible region. All other predictor variables are held at average values found in this study.

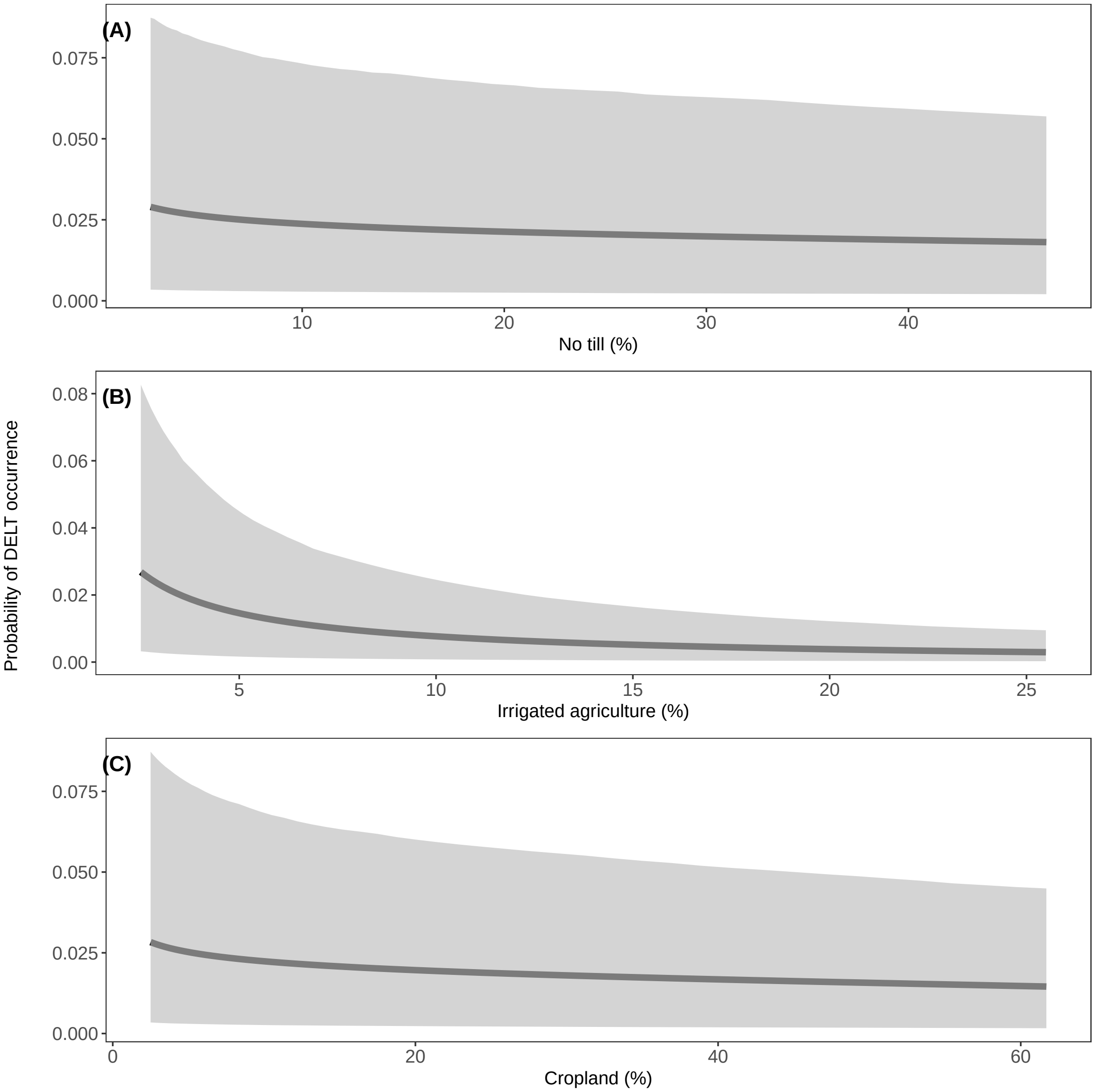

Figure A6: Predicted probability of DELT occurrence across all fish species sampled in the Chesapeake Bay watershed, USA (modeled using equation (2)) as a function of land on which no tillage practices were used (A), irrigated agriculture (B), and cropland (C). Solid line is posterior mean and shaded area is 90% credible region. All other predictor variables are held at average values found in this study.

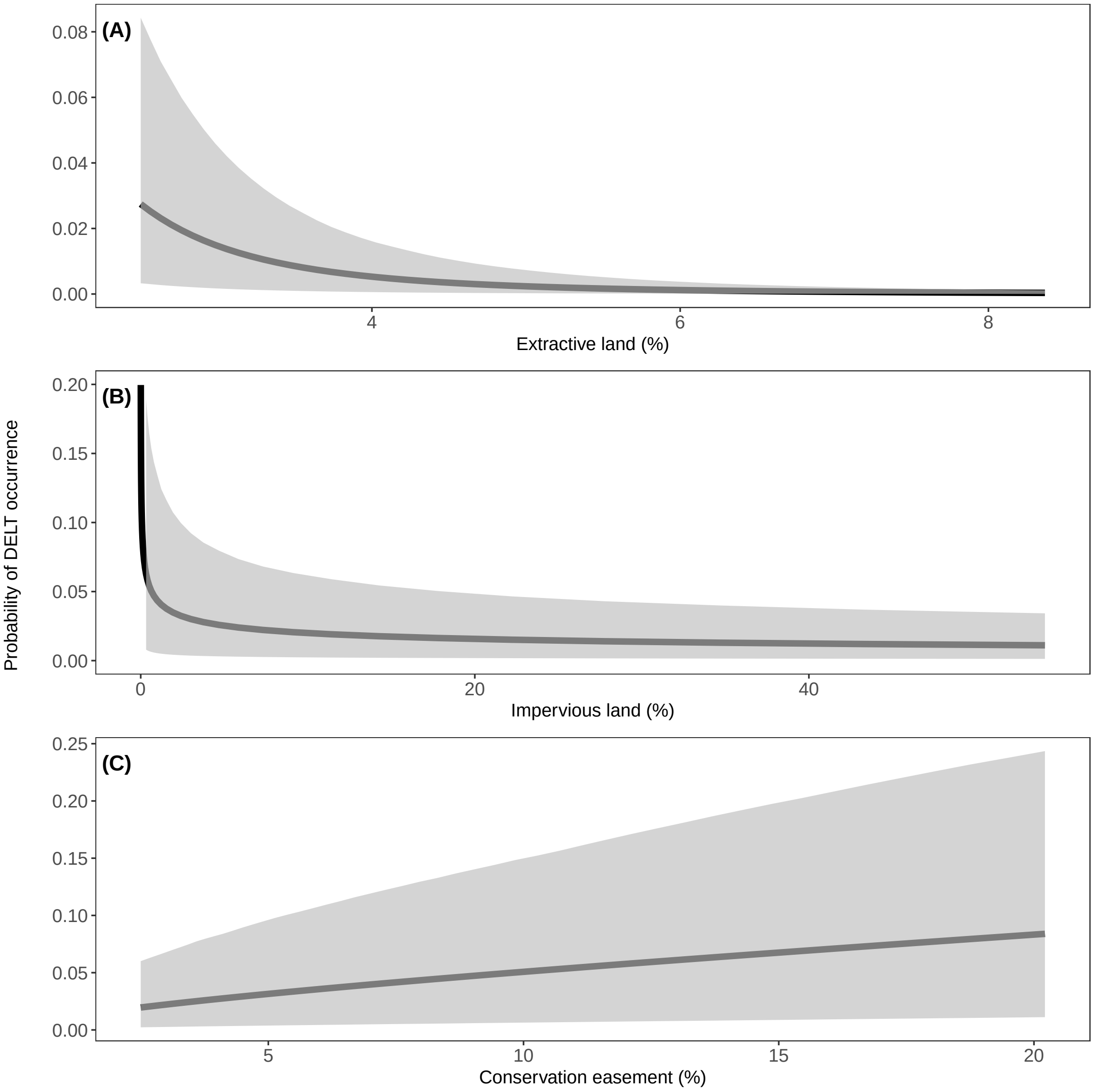

Figure A7: Predicted probability of DELT occurrence across all fish species sampled in the Chesapeake Bay watershed, USA (modeled using equation (2)) as a function of extractive land (A), impervious land (B), and land under a conservation easements (C). Solid line is posterior mean and shaded area is 90% credible region. All other predictor variables are held at average values found in this study.

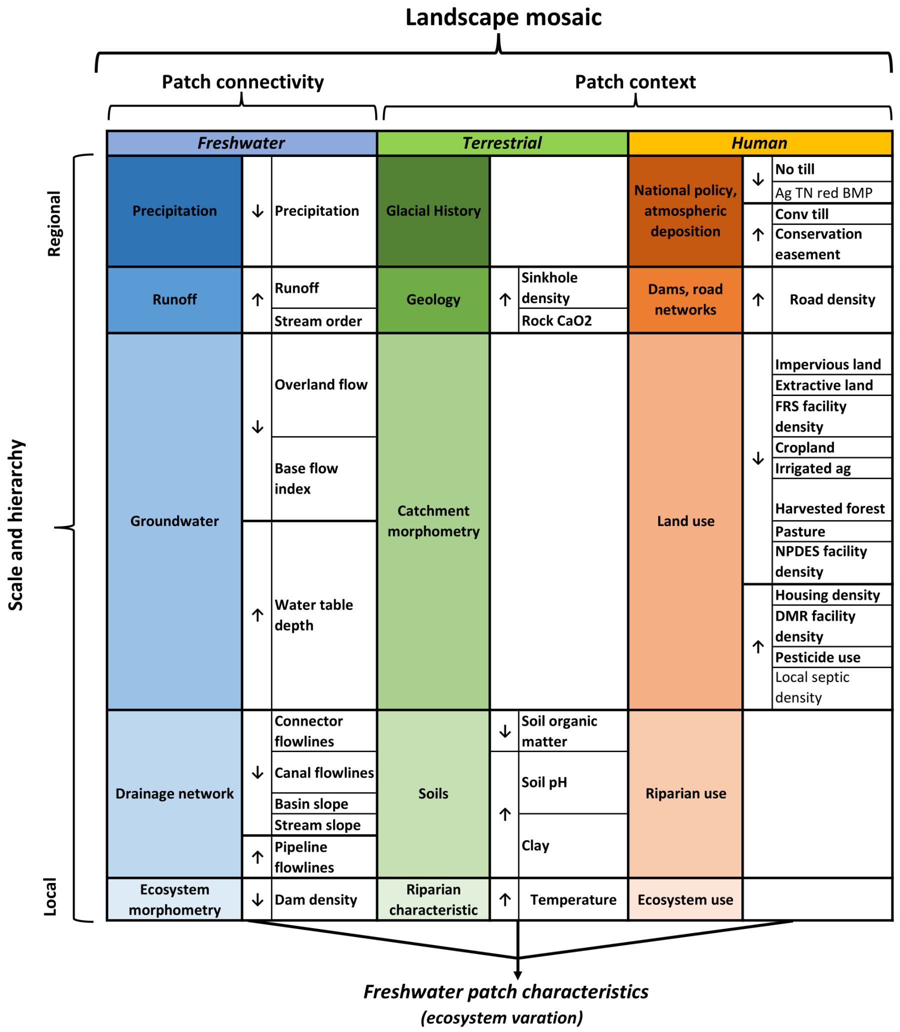

Figure A8: Significant landscape predictors of DELT occurrence across all fish species in the Chesapeake Bay watershed, USA (modeled using equation (2)), organized in a patch-mosaic landscape. Bolded predictors have 90% confidence intervals that did not include the value zero and non-bolded predictors have a posterior probability that the estimated parameter is in the direction of the posterior mean (either positive or negative) > 0.90. The up arrows indicate increased probability of DELT occurrence with increases in the predictor(s) and the down arrows indicate declined probability of DELT occurrence with increases in the predictor(s).

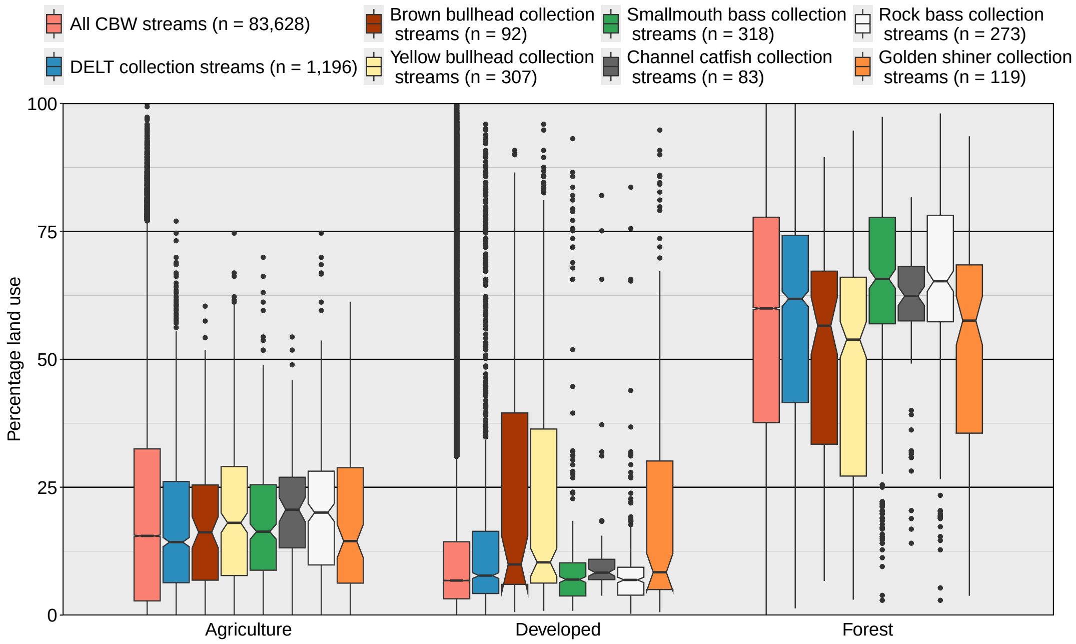

Figure A9: Notched box plots of percentages of major land use in all streams of the Chesapeake Bay watershed (CBW), collection streams included in the fish DELT model across all species (modeled using equation (1)) in blue, and collection streams of the six individual fish species analyzed in species-specific DELT models (modeled using equation (2), except without a species random effect).Within each boxplot, the box represents the interquartile range (IQR), containing the middle 50% of the data with the lower and upper edges indicating the first (Q1) and third (Q3) quartiles, respectively. The line inside the box shows the median (Q2). The notch in the box provides an approximate 95% confidence interval for the median; non-overlapping notches suggest a statistically significant difference in medians between groups. Individual points plotted beyond the whiskers represent potential outliers (Wickham 2016).

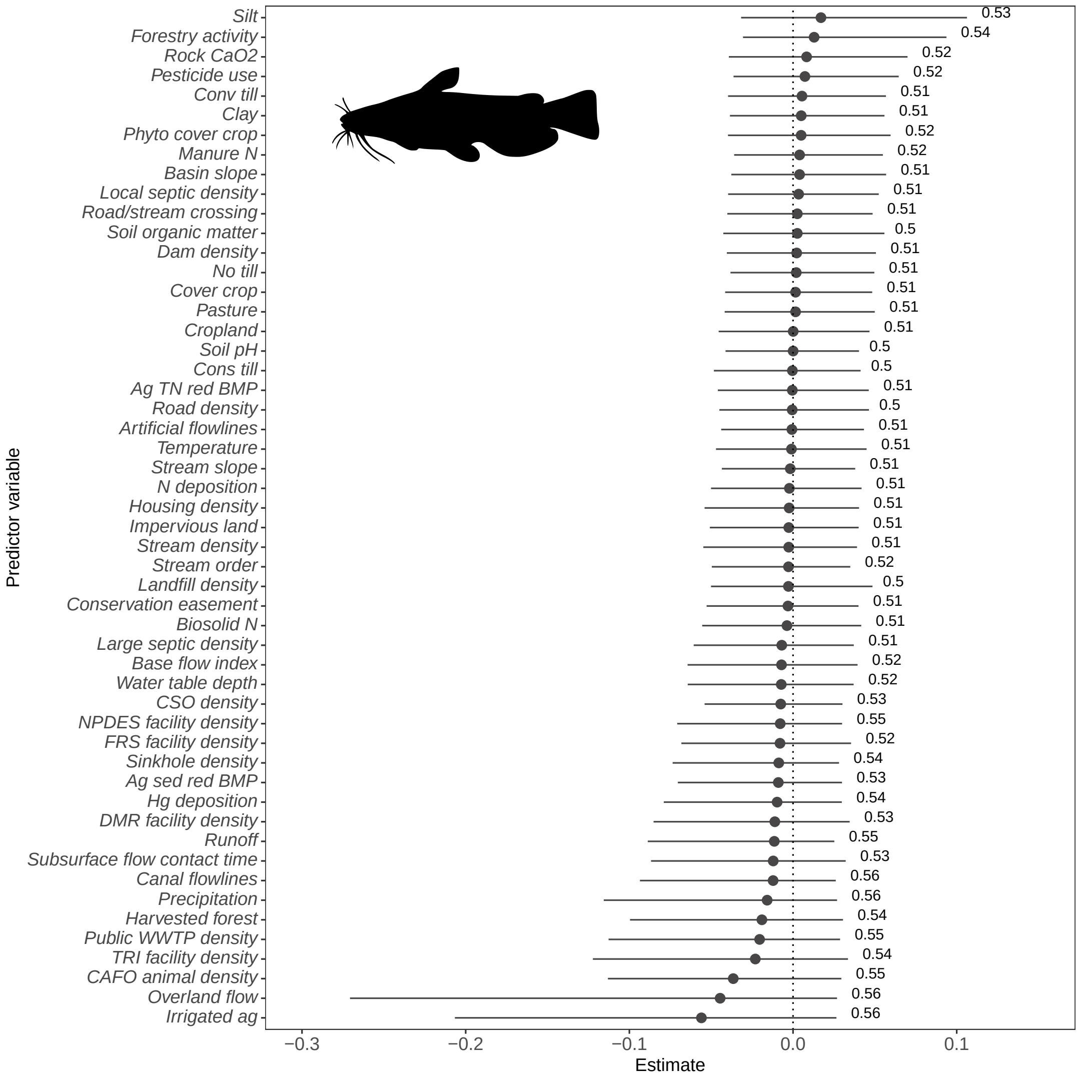

Figure A10: The effects of landscape predictors on the probability of DELT occurrence for Brown Bullhead *Ameiurus nebulosus* in the Chesapeake Bay watershed, USA (modeled using equation (2), except without a species random effect). Estimated effects (β) are shown on the x-axis as posterior means (circles) and 90% credible intervals (horizontal bars). Predictors are all shown in grey and have 90% credible intervals that include the value zero. The posterior probability for each predictor is displayed as a numerical value. Fish silhouette is from the R system rphylopic package (R Core Team 2024, Gearty and Jones 2023).

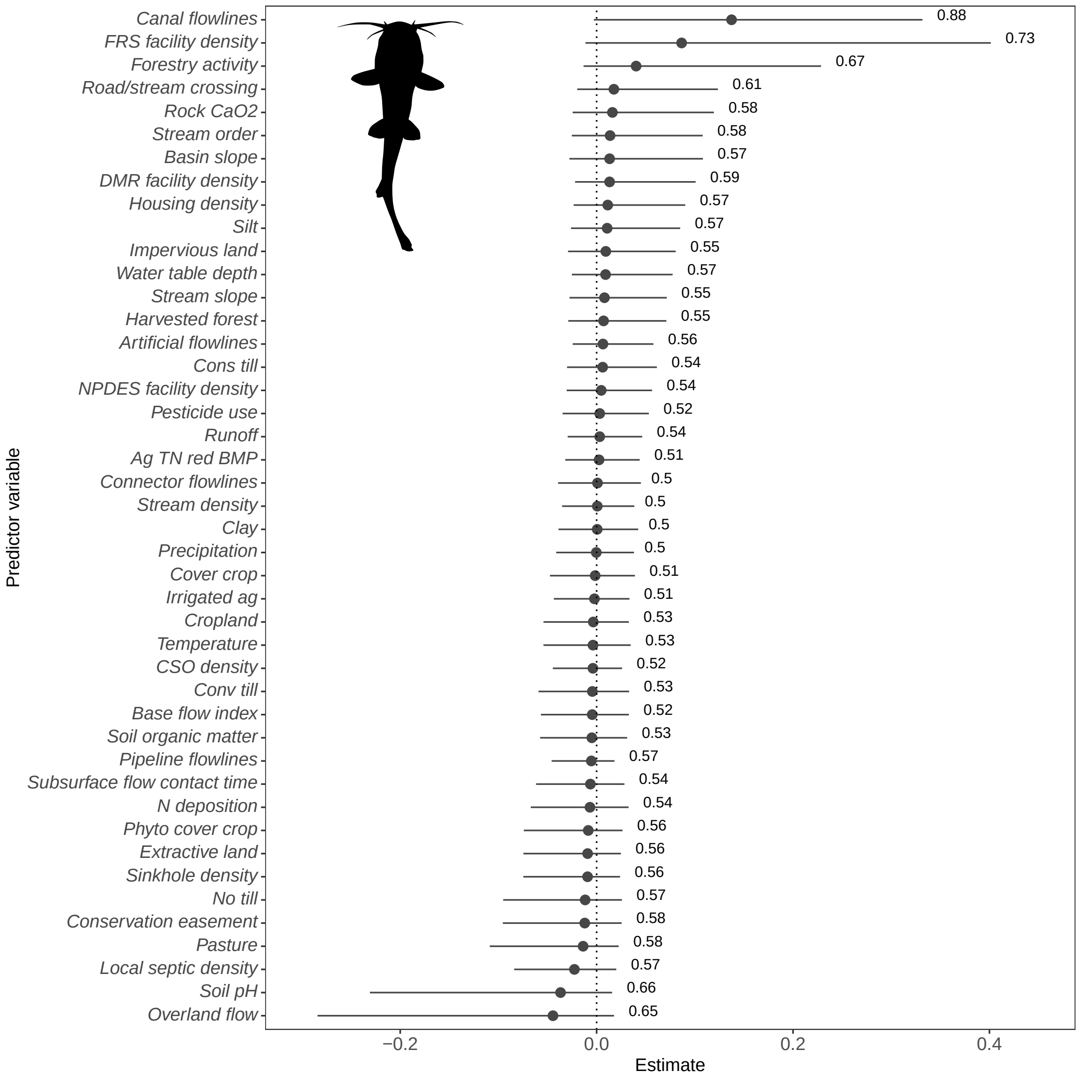

Figure A11: The effects of landscape predictors on the probability of DELT occurrence for Yellow Bullhead *Ameiurus natalis* in the Chesapeake Bay watershed, USA (modeled using equation (2), except without a species random effect). Estimated effects (β) are shown on the x-axis as posterior means (circles) and 90% credible intervals (horizontal bars). Predictors are all shown in grey have 90% credible intervals that include the value zero. The posterior probability for each predictor is displayed as a numerical value. Fish silhouette is from the R system rphylopic package (R Core Team 2024, Gearty and Jones 2023).

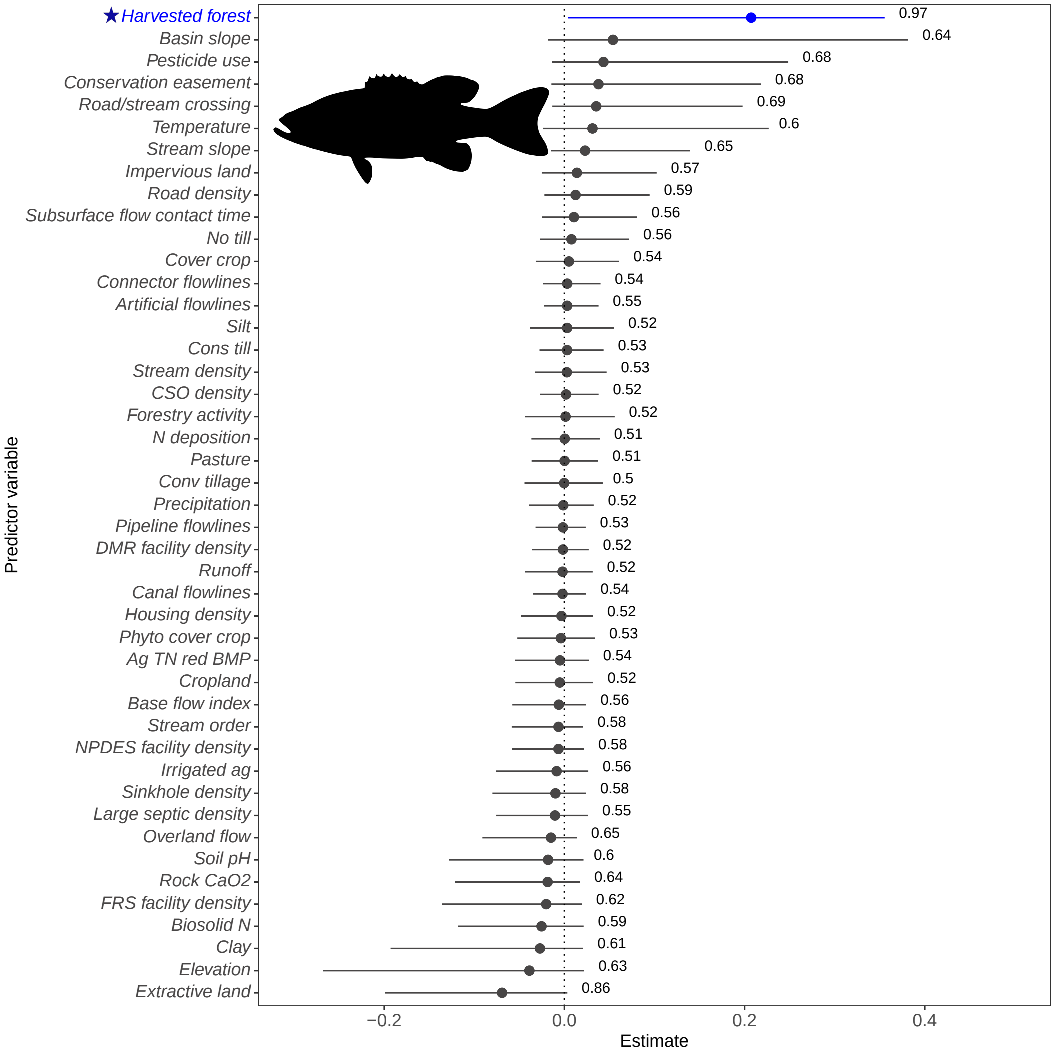

Figure A12: The effects of landscape predictors on the probability of DELT occurrence for Smallmouth Bass *Micropterus dolomieu* in the Chesapeake Bay watershed, USA (modeled using equation (2), except without a species random effect). Estimated effects (β) are shown on the x-axis as posterior means (circles) and 90% credible intervals (horizontal bars). The predictors marked with a star and highlighted in blue has a 90% credible interval that did not include the value zero and predictors shown in grey have 90% credible intervals that include the value zero. The posterior probability for each predictor is displayed as a numerical value. Fish silhouette is from the R system rphylopic package (R Core Team 2024, Gearty and Jones 2023).

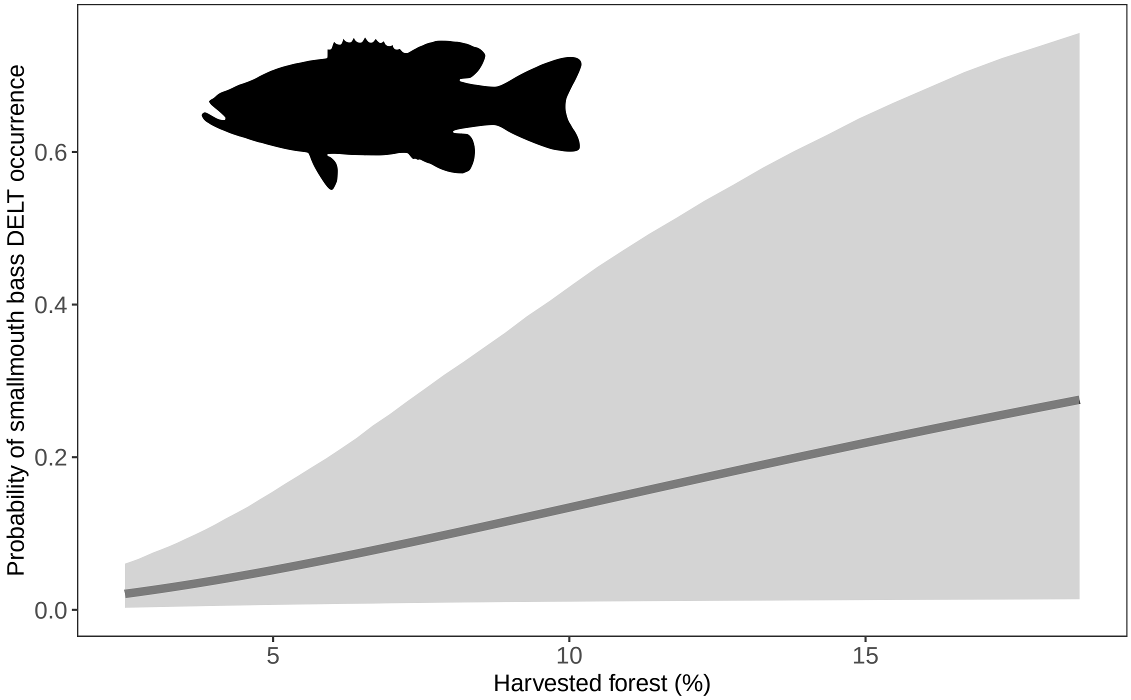

Figure A13: Predicted probability of DELT occurrence for smallmouth bass (*Micropterus dolomieu*) sampled in the Chesapeake Bay watershed, USA as a function of harvested forest (modeled using equation (2), except without a species random effect). Solid line is posterior mean and shaded area is 90% credible region. All other predictor variables are held at average values. Fish silhouette is from the R system rphylopic package (R Core Team 2024, Gearty and Jones 2023).

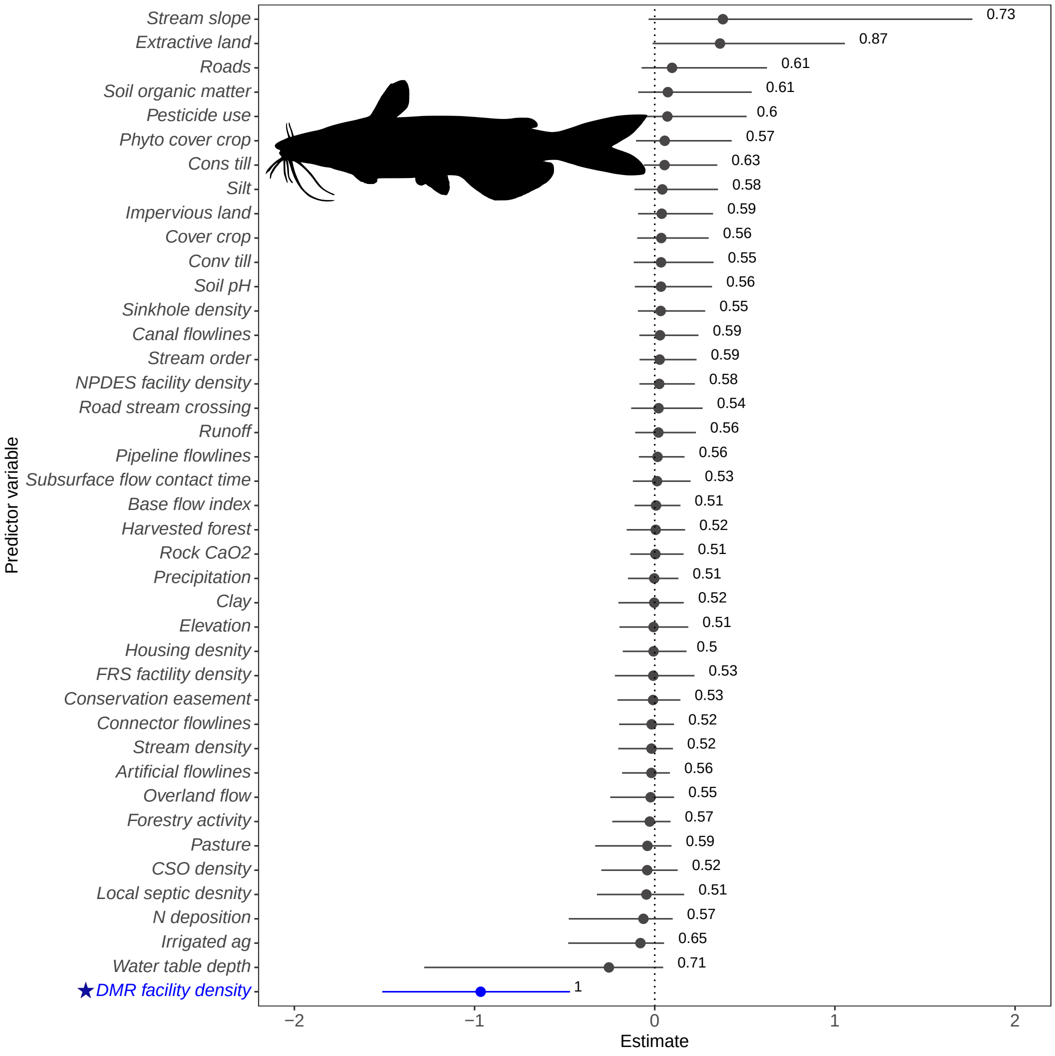

Figure A14: The effects of landscape predictors on the probability of DELT occurrence for Channel Catfish *Ictalurus punctatus* in the Chesapeake Bay watershed, USA (modeled using equation (2), except without a species random effect). Estimated effects (β) are shown on the x-axis as posterior means (circles) and 90% credible intervals (horizontal bars). The predictor marked with a star and highlighted in blue has a 90% credible interval that did not include the value zero and predictors shown in grey have 90% credible intervals that include the value zero. The posterior probability for each predictor is displayed as a numerical value. Fish silhouette is from the R system rphylopic package (R Core Team 2024, Gearty and Jones 2023).

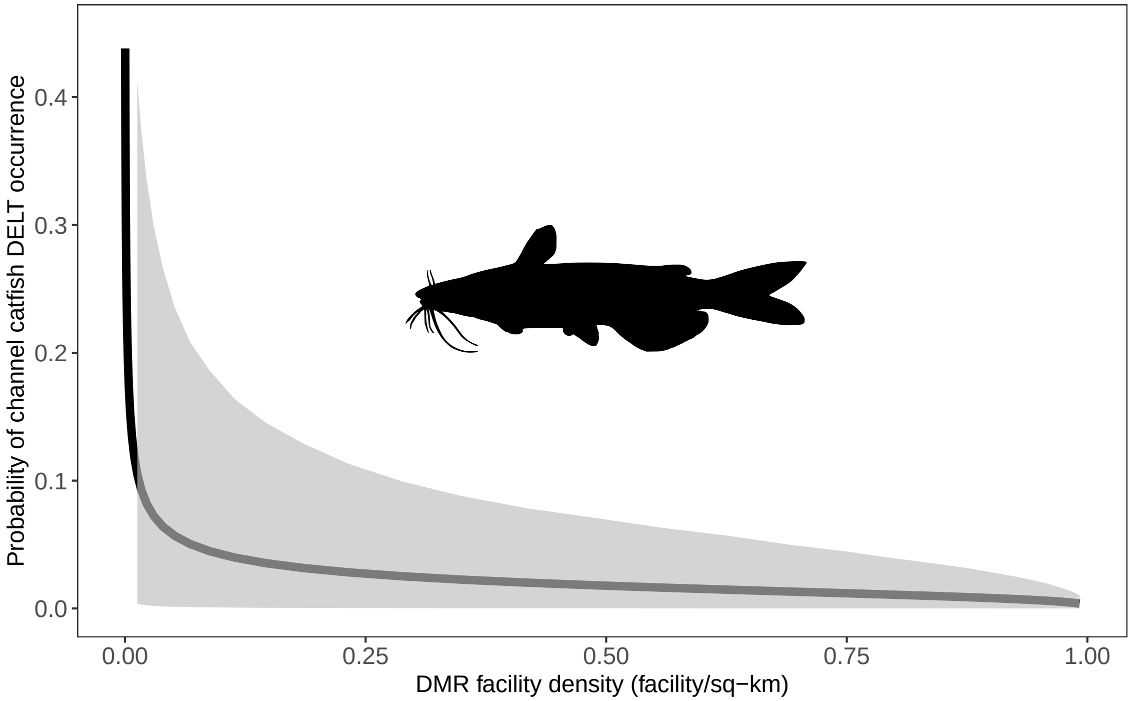

Figure A15: Predicted probability of DELT occurrence for Channel Catfish *Ictalurus punctatus* sampled in the Chesapeake Bay watershed, USA as a function of DMR facility density (modeled using equation (2), except without a species random effect). Solid line is posterior mean and shaded area is 90% credible region. All other predictor variables are held at average values. For predictor definitions refer to Table A5. Fish silhouette is from the R system rphylopic package (R Core Team 2024, Gearty and Jones 2023).

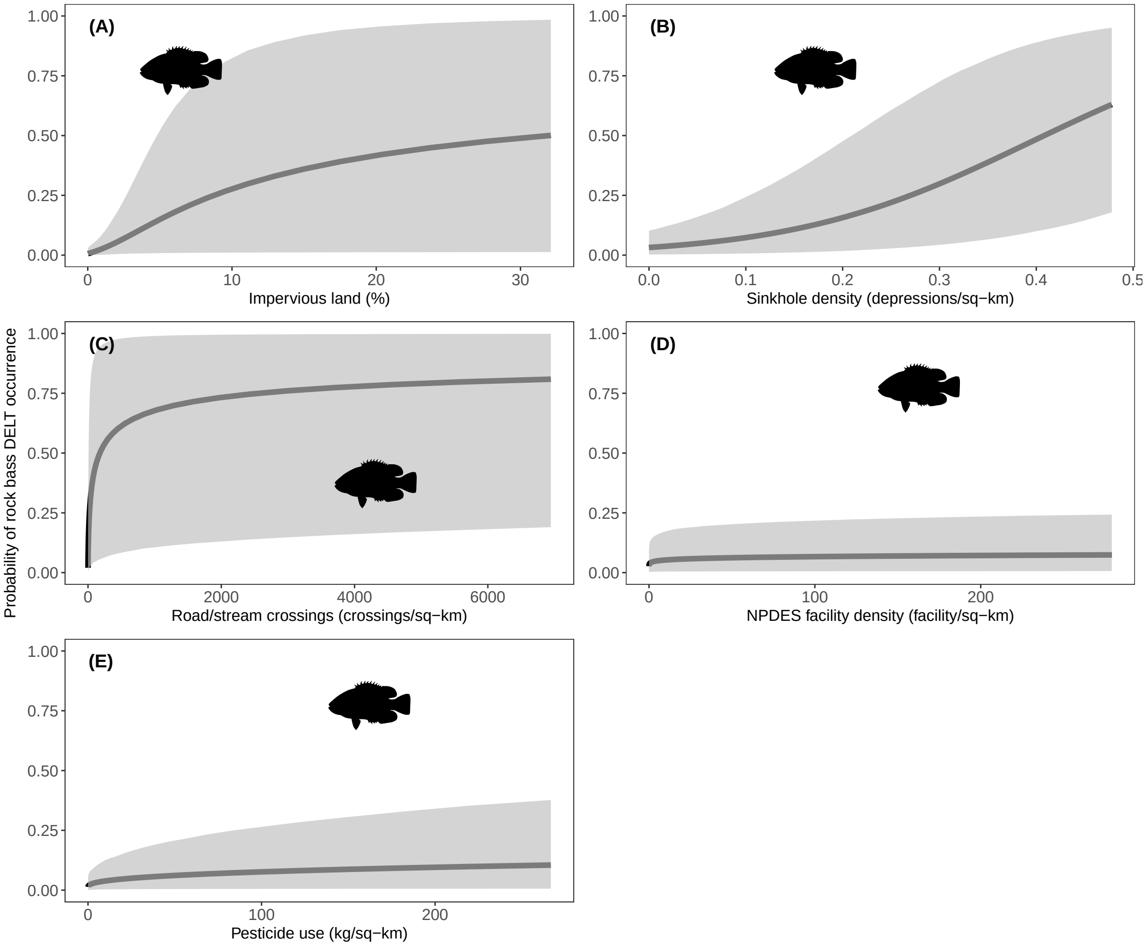

Figure A16: Predicted probability of DELT occurrence for Rock Bass *Ambloplites rupestris* sampled in the Chesapeake Bay watershed, USA as a function of impervious land (A), sinkhole density (B), road/stream crossings (C), NPDES facility density (D), and estimated pesticide use (E); (modeled using equation (2), except without a species random effect). Solid line is posterior mean and shaded area is 90% credible region. All other predictor variables are held at average values. For predictor definitions refer to Table A5. Fish silhouette is from the R system rphylopic package (R Core Team 2024, Gearty and Jones 2023).

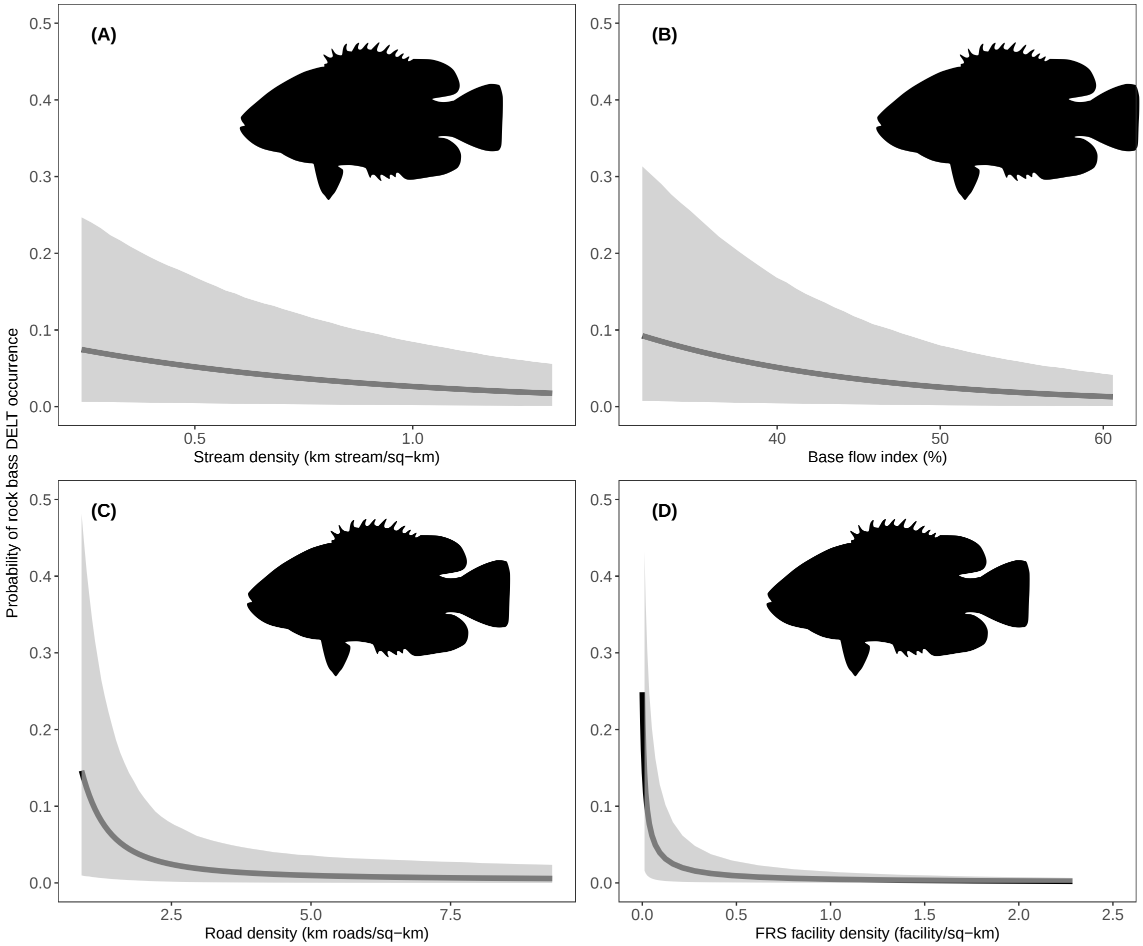

Figure A17: Predicted probability of DELT occurrence for Rock Bass *Ambloplites rupestris* sampled in the Chesapeake Bay watershed, USA as a function of stream density (A), base flow index (B), road density (C), and FRS facility density (D); (modeled using equation (2), except without a species random effect). Solid line is posterior mean and shaded area is 90% credible region. All other predictor variables are held at average values. For predictor definitions refer to Table A5. Fish silhouette is from the R system rphylopic package (R Core Team 2024, Gearty and Jones 2023).

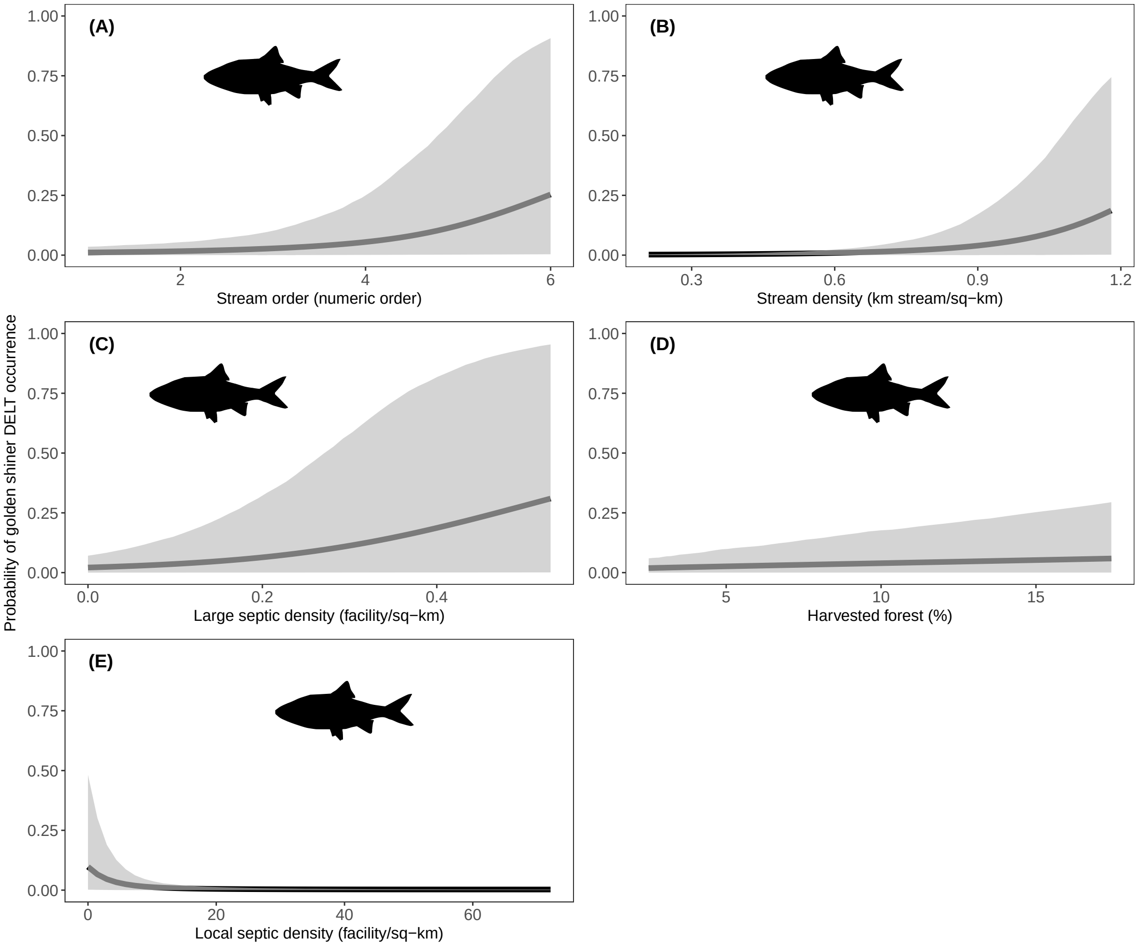

Figure A18: Predicted probability of DELT occurrence for Golden Shiner *Notemigonus crysoleucas* sampled in the Chesapeake Bay watershed, USA as a function of stream order (A), stream density (B), large septic density (C), harvested forest (D), and local septic density (E); (modeled using equation (2), except without a species random effect). Solid line is posterior mean and shaded area is 90% credible region. All other predictor variables are held at average values. For predictor definitions refer to Table A5. Fish silhouette is from the R system rphylopic package (R Core Team 2024, Gearty and Jones 2023).
